# A bibliometric analysis of endoplasmic reticulum stress and atherosclerosis

**DOI:** 10.3389/fphys.2024.1392454

**Published:** 2024-06-11

**Authors:** Xinyu Huang, Feng Jiang, Yongbo Ma, Kunpeng Zhu, Zhenyuan Wang, Zhen Hua, Jie Yu, Lei Zhang

**Affiliations:** ^1^ Shandong University of Traditional Chinese Medicine, Shandon, China; ^2^ Affiliated Hospital of Shandong University of Traditional Chinese Medicine, Shandon, China

**Keywords:** endoplasmic reticulum stress, atherosclerosis, knowledge-map, Citespace, VOSviewer, bibliometrics

## Abstract

The mechanisms underlying the occurrence and development of atherosclerosis (AS) are diverse, among which endoplasmic reticulum stress (ERS) is an important mechanism that should not be overlooked. However, up to now, there has been no bibliometric study on the relationship between ERS and AS. To understand the research progress in ERS and AS, this paper conducted a statistical analysis of publications in this field using bibliometrics. A total of 1,035 records were retrieved from the Web of Science Core Collection. CiteSpace, VOSviewer, and the R package “bibliometric” were used to analyze the spatiotemporal distribution, countries, authors, institutions, journals, references, and keywords of the literature, and to present the basic information of this field through visualized maps, as well as determine the collaboration relationships among researchers in this field. This field has gradually developed and stabilized over the past 20 years. The current research hotspots in this field mainly include the relationship between ERS and AS-related cells, the mechanisms by which ERS promotes AS, related diseases, and associated cytokines, etc. Vascular calcification, endothelial dysfunction, NLRP3 inflammasome, and heart failure represent the frontier research in this field and are becoming new research hotspots. It is hoped that this study will provide new insights for research and clinical work in the field of ERS and AS.

## 1 Introduction

Atherosclerosis (AS) is a chronic inflammatory vascular disease in which lipid or fibrous plaques deposit on the inner lining of arteries, causing narrowing of the affected vessels, blood flow obstruction, and ischemia of related tissues (Libby et al., 2019). Narrowing of blood vessels coupled with the rupture of unstable plaques can lead to platelet aggregation, thrombosis formation, and acute cardiovascular and cerebrovascular diseases. Cardiovascular disease is the leading cause of death worldwide (Benjamin et al., 2017), and coronary heart disease is a major component of cardiovascular disease. An epidemiological survey conducted in the United States showed that more than 17 million people died from cardiovascular disease in 2015, accounting for 31% of all deaths worldwide, and an estimated 7.4 million people died from coronary heart disease (Organization, 2011). Moreover, over 75% of deaths from cardiovascular disease occur in low- and middle-income countries worldwide (Organization, 2011). The high disability rates associated with cardiovascular disease also impose a heavy economic burden on many developing countries.

**FIGURE 1:**
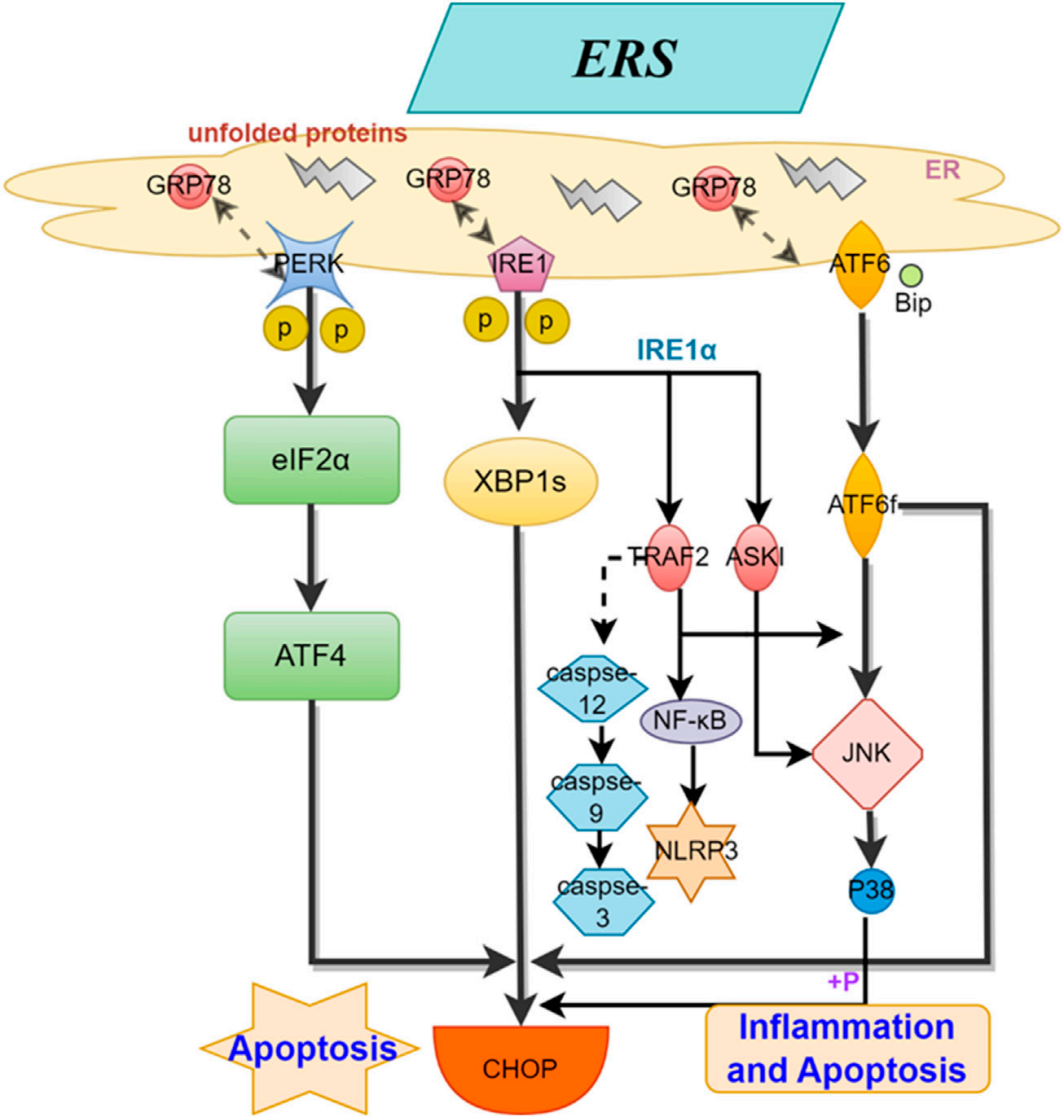
Mechanisms of ERS for AS. This figure illustrates the process by which the three stress sensors, IRE1, PERK, and ATF6, located on the ER membrane, induce inflammation and cell apoptosis through distinct mechanisms.

The endoplasmic reticulum (ER) is an important organelle in eukaryotic cells that controls protein quality and is responsible for protein synthesis, folding, and transport, as well as participating in calcium storage. Research has shown that ERS is a key factor in the development of AS (Tabas, 2010). The ER membrane contains three types of transmembrane proteins: activating transcription factor 6 (ATF6), double-stranded RNA-dependent protein kinase R-like ER kinase (PERK), and inositol-requiring enzyme 1 (IRE1) (Ron and Walter, 2007; Adolph et al., 2012; Hetz, 2012). When ERS is enhanced, these sensors can recognize and initiate a series of complex adaptive responses to restore ER homeostasis and function, known as the unfolded protein response (UPR). UPR can provide cellular protection when moderately activated, but if the stress exceeds the compensatory capacity of UPR, such as under oxidative stress, hypoxia, or inflammatory reactions, it can disrupt ER homeostasis, enhance ERS, lead to misfolding and accumulation of unfolded proteins in the lumen, along with disturbances in calcium balance. This can activate inflammatory and apoptotic pathways, resulting in cellular and endothelial dysfunction, accelerating plaque formation, and the progression of AS (Lawrence de Koning et al., 2003; Yang et al., 2020). The specific mechanism (shown in Figure 1) involves the separation of the ER membrane-located stress sensors IRE1, PERK, and ATF6 from their protein partner Bip. IRE1 splices XBP1 mRNA to generate spliced XBP1 and recruits apoptosis signal-regulating kinase 1 (ASK1) and TNF receptor-associated factor 2 (TRAF2) (Zhu and Lee, 2015; Lebeaupin et al., 2018), leading to the activation of JNK and NF-kB, inducing inflammation and cell apoptosis. Activated PERK phosphorylates the eukaryotic initiation factor 2α (eIF2α), indirectly inhibiting protein synthesis and RNA transcription (Yang et al., 2020), while enhanced phosphorylated eIF2α promotes ATF4 mRNA translation, upregulating the transcription factor C/EBP homologous protein (CHOP), inducing gene expression involved in autophagy, oxidative stress, and apoptosis (Ren et al., 2021). Unfolded proteins in the ER lumen activate the JNK and CHOP pathways after the dissociation of ATF6 and heavy chain-binding protein (Bip)/GRP78(11). Under sustained ER stress conditions, the activated CHOP/GADD153, Caspase-12, and JNK pathways participate in inducing cell apoptosis. Additionally, the activation of the TRAF2-mediated NF-κB pathway induces the production of NLRP3 inflammasome (NLRP3) and inflammatory factors such as interleukins, triggering an inflammatory response (Bravo et al., 2011; Oakes and Papa, 2015). All of these findings indicate the significant role of ERS in the formation and progression of atherosclerosis.

Bibliometrics emerged in the early 20th century and developed into an independent discipline in 1969. It has been widely applied in literature analysis. Bibliometrics provides quantitative and qualitative methods for investigating published literature in various fields. By analyzing specific information such as authors, countries, institutions, journals, and references of published literature, bibliometrics enables us to obtain information about the development status, distribution patterns, and research hotspots in relevant fields. It has become one of the popular techniques for evaluating the credibility, quality, and impact of academic work (Ellegaard and Wallin, 2015; Rondanelli et al., 2016). Quantitative analysis of literature-related data combined with visual presentations can make research results more intuitive. A search of literature related to ERS and AS reveals that although numerous studies are exploring this field from different perspectives, the use of bibliometric methods to analyze the research status in this field is lacking.

## 2 Methods

### 2.1 Data collection

**FIGURE 2:**
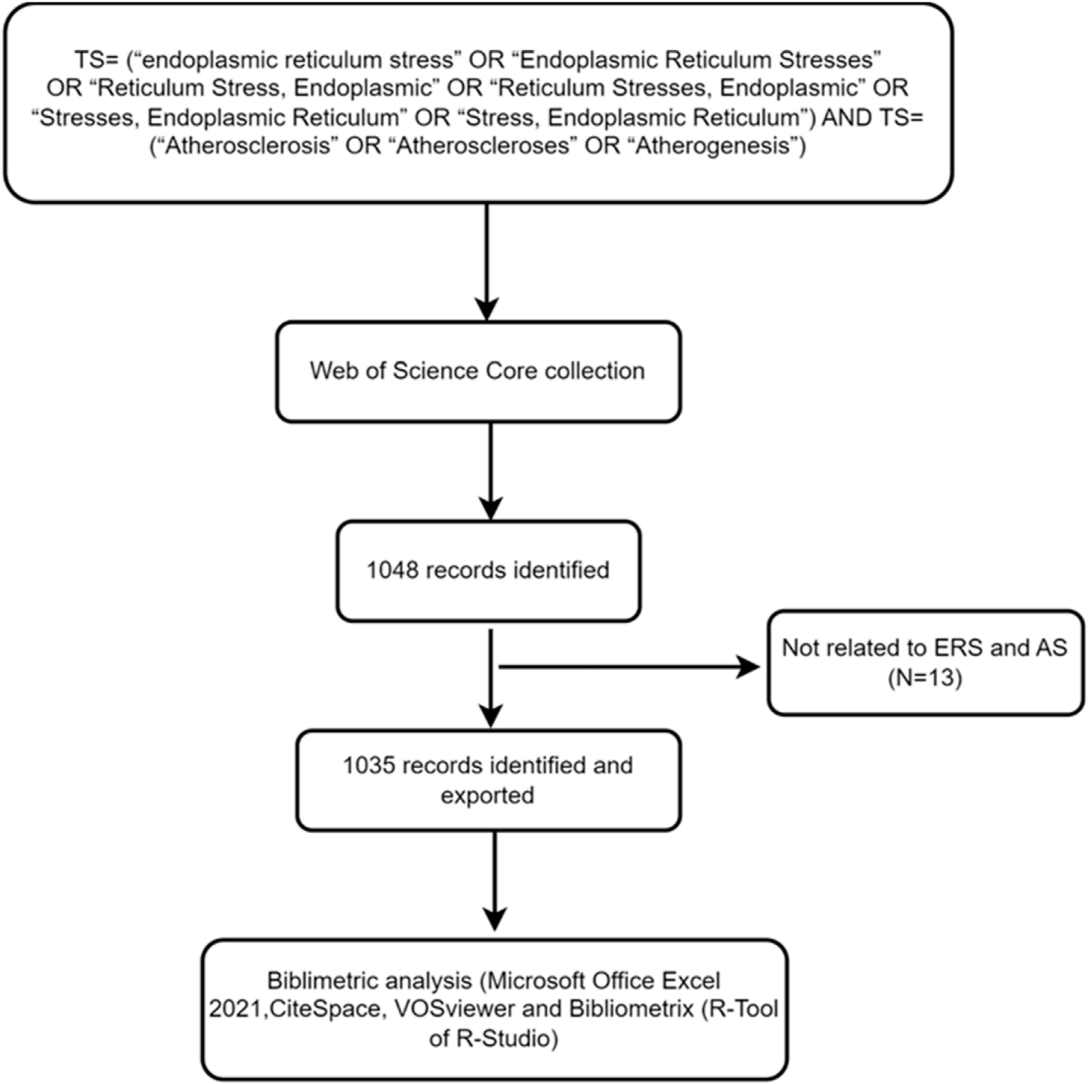
Flowchart illustrating the search strategy and selection process in ERS and AS.

Web of Science is a globally trusted and authoritative scientific citation database that covers multiple fields including natural sciences, engineering technology, biomedical sciences, social sciences, arts, and humanities. To ensure the coverage and authority of the data, the Web of Science was selected as the data source, the indexes were selected as SSCI and SCIE, the search strategy selected was TS= (“endoplasmic reticulum stress” OR “Endoplasmic Reticulum Stresses” OR “Reticulum Stress, Endoplasmic” OR “Reticulum Stresses, Endoplasmic” OR “Stresses, Endoplasmic Reticulum” OR “Stress, Endoplasmic Reticulum”) AND TS= (“Atherosclerosis” OR “Atheroscleroses” OR “Atherogenesis”). To avoid deviations caused by daily data updates, the time span was set from 1 January 2000 to 1 February 2023. A total of 1,048 documents were obtained, and 1,035 documents were finally used in the study. The search results were exported with “Plain Text file” and the record content chose “Full Record and Cited Reference”, and stored in download_*.txt format. The flowchart was shown in Figure 2.

### 2.2 Data analysis and visualization

In this study, CiteSpace, VOSviewer and the R package of " bibliometric” were applied to collect information on ERS and AS and draw knowledge maps. These software programs have complementary advantages. CiteSpace uses a set-theoretic data standardization method to measure the similarity of knowledge units. Similarity algorithms are used to obtain Timezone and Timeline views within time slices, which clearly outline the evolution of knowledge in this field over time, enabling understanding of the development dynamics and exploration of new research hotspots (Chen, 2006). VOSviewer uses a probability-based data standardization method and provides multiple visualizations in co-authorship, co-citation, and keyword sections, including Network Visualization, Overlay Visualization, and Density Visualization, with the advantages of simple operation and beautiful images (van Eck and Waltman, 2010). R language is a programming language and operating environment used for statistical analysis, graphical reports, and reporting. “bibliometric” is an R language software package developed based on bibliometrics. It uses its powerful computing ability to extract multiple analysis indicators of literature and can achieve visual analysis of bibliometric analysis (Aria et al., 2017). Firstly, we used visualized maps of annual publications, countries, authors, institutions, and journal publications to intuitively display the basic output of this field. Secondly, based on highly co-cited journals and literature, we presented the knowledge base of this field. Finally, based on co-occurrence graphs of keywords (including keyword clustering, timeline, and highlight maps), we showed changes in research hotspots in this field and identified and discovered new hotspots.

## 3 Results

### 3.1 Temporal distribution map of the literature

**FIGURE 3:**
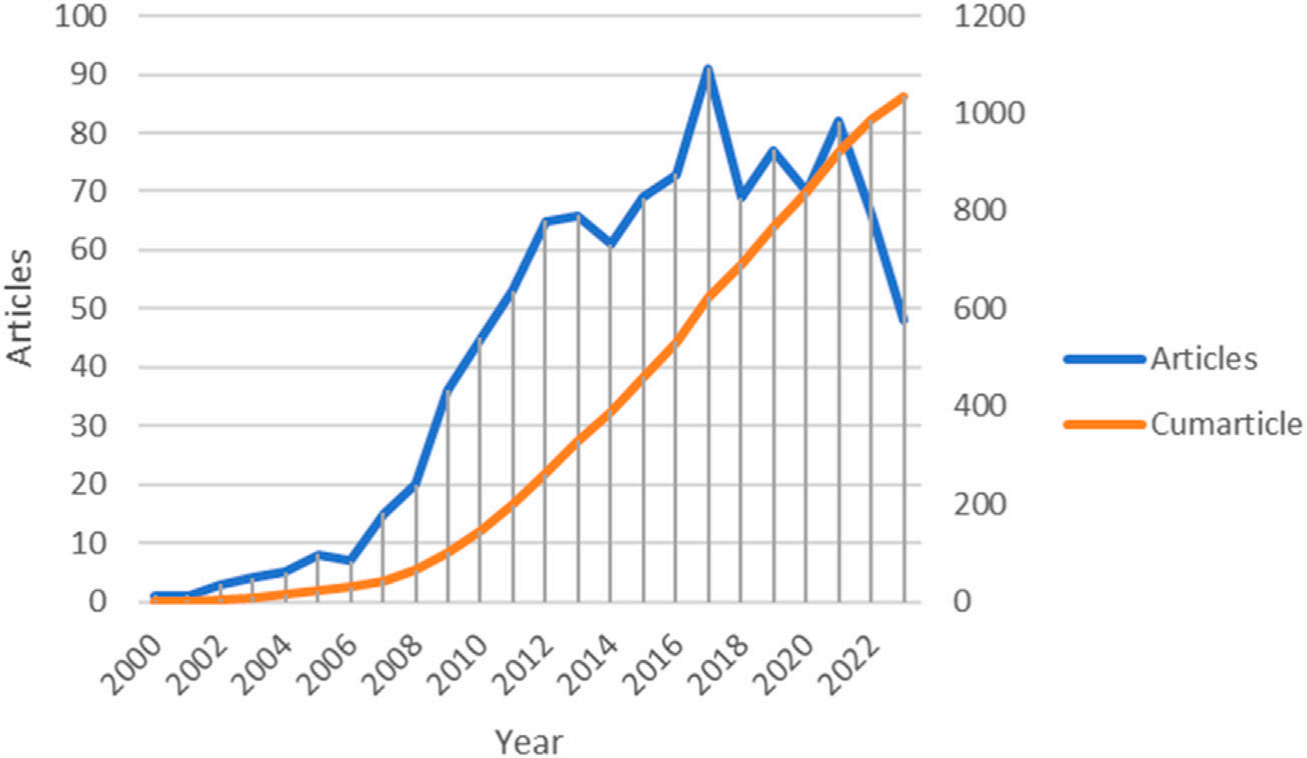
Trends in the growth of publications and the number of cumarticles.

From January 2000 to December 2023, a total of 1,035 publications were published in this field. Figure 3 shows the trend of literature production in this field over time. The first relevant literature on ERS and AS appeared in 2000, and since then, research on ERS and AS has shown a steady and significant increase. It reached its peak in 2017 with 91 publications. After that, the research intensity on ERS and AS slightly declined but still exhibited a fluctuating upward trend. As the statistics for 2023 are not yet complete, there is a downward trend starting from 2021, and the number of publications in 2023 reached its lowest point at 48.

### 3.2 Distribution of countries/regions

**FIGURE 4:**
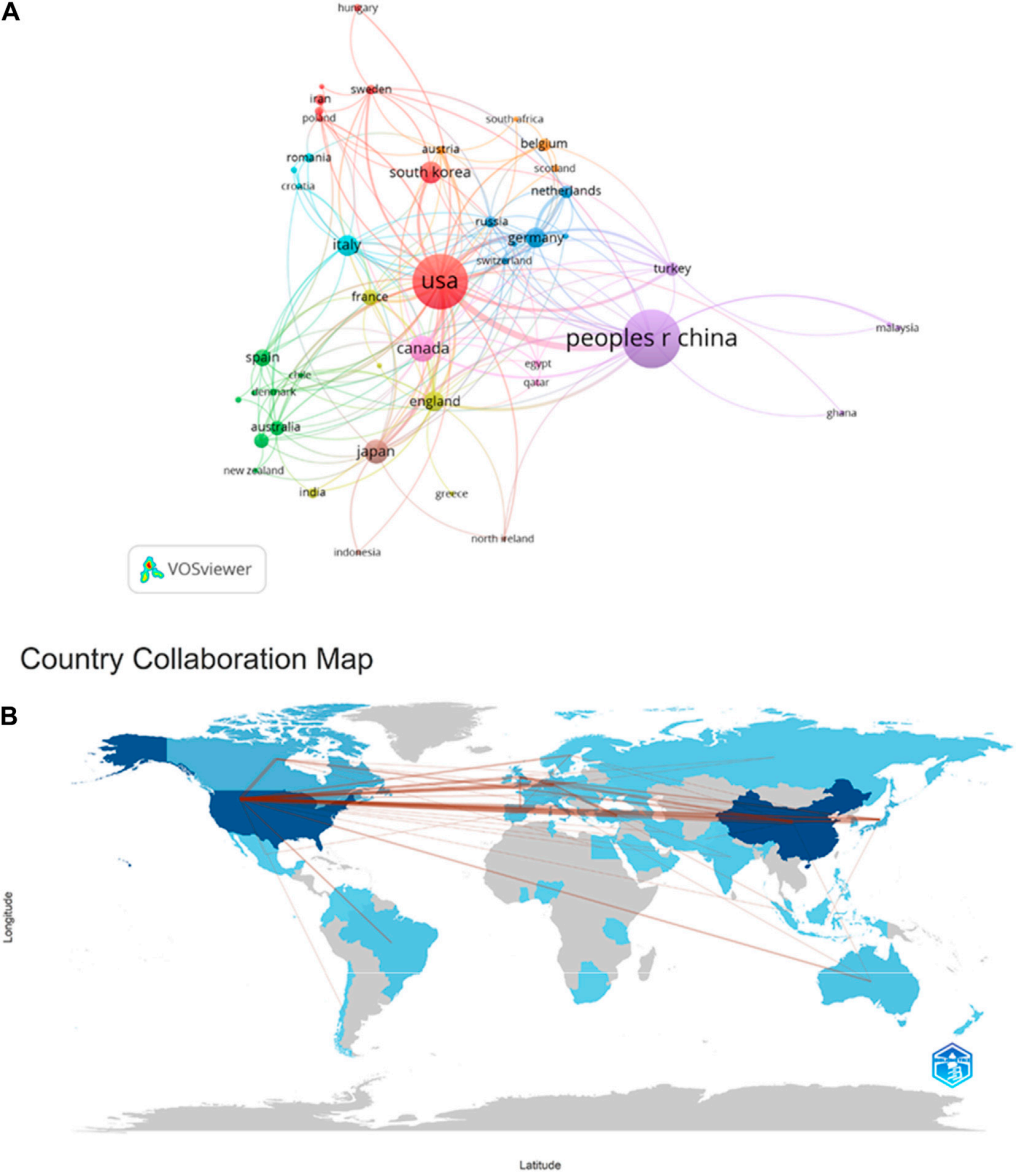
Cooperation map of countries/regions in ERS and AS. **(A)** A visual map for VOSviewer network. **(B)** Countries/regions involved in ERS and AS research. The links between countries/regions indicate their collaborations and connections.

As shown in Figure 4 and Table 1, China has the highest number of publications, with a total of 348 papers. The following countries are the United States, Canada, Japan, and South Korea, with 318, 70, 62, and 50 publications respectively, accounting for 33.62%, 30.72%, 6.76%, 5.99%, and 4.83% of the total publication count, surpassing 80% of the total. This suggests that these countries have made significant contributions to the field, while also highlighting the uneven development of research in this domain across different countries/regions. Among the top ten countries in terms of publication count displayed in the table, six countries have a centrality score exceeding 0.1: PEOPLES R CHINA (0.26), United States (0.51), Canada (0.1), ITALY (0.14), ENGLAND (0.27), and FRANCE (0.17). These scores indicate that these six countries hold an important position in research on ERS and AS. It is noteworthy that the United States has the highest citation count, reaching 36,719, followed by China and Canada with 9,528 and 5,052 citations respectively. This highlights the high research value of literature from these countries, which has garnered attention from numerous scholars. VOSviewer parameters were set as follows: Methods (Linlog/modularity) and a minimum number of country documents: 5. The obtained results were retrieved from 62 countries, with 30 meeting the thresholds.

**TABLE 1:** Top 10 most productive countries/regions in ERS and AS.

Rank	Countries/Regions	Record count	% of 1,035	Average per Item	Citations	Total link strength	Centrality
1	PEOPLES R CHINA	348	33.66	27.40	9,528	78	0.26
2	United States	318	30.72	115.47	36,719	170	0.51
3	Canada	70	6.76	72.17	5,052	44	0.1
4	Japan	62	5.99	74.03	4,590	37	0.08
5	South korea	50	4.83	35.28	1764	15	0.01
6	Italy	48	4.64	54.21	2,602	28	0.14
7	Germany	44	4.25	109.5	4,818	54	0.05
8	England	40	3.86	121.45	4,858	48	0.27
9	Spain	31	2.99	52.65	1,632	12	0.06
10	France	27	2.61	75.37	2035	31	0.17

According to the analysis of collaborative authors, VOSviewer divided countries into different collaboration clusters, distinguished by different colored nodes. The size of the nodes represents the number of publications, and the thickness of the lines indicates the number of connections between nodes (Figure 3A). Figure 3A shows that countries that collaborate more with the United States include China, Canada, the United Kingdom, Japan, France, Germany, Italy, South Korea, and others. Countries that collaborate more with China include the United States, Canada, the United Kingdom, Turkey, and others. Countries that collaborate more with Canada include China, the United States, the United Kingdom, France, and others. Figure 3B displays a collaboration map of authors in ERS and AS based on their countries/regions. The darker the color, the higher the publication count. The lines represent collaborative relationships between countries, and the line thickness represents the strength of collaboration. Interestingly, the more frequent the contributions between countries, the greater the output, as exemplified by China and the United States, where the figure showed that the lines crossing them are numerous and thick, suggesting that China and the United States play an important role in the development of cooperation in this field. Thus China and the United States are both the countries that produce the most output and work closely with Canada, France, Germany, and Japan.

### 3.3 Distribution of authors and research institutions

Analyzing the literature authors allows us to identify the representative scholars and core research forces in this field. According to Price’s Law, the minimum publication requirement for core authors in a particular field is given by m = 0.749 * √nmax (where nmax is the maximum number of publications by any author)≈4.43. Therefore, authors with more than four publications (including 4) were identified as core authors in this field, totaling 127 core authors with a combined publication count of 706 papers, accounting for 68.2% of the total publication count. This meets Price’s criterion of reaching 50% of the total, indicating that the ERS and AS fields have formed a relatively stable collaborative community.

As shown in Table 2, 5 authors have published more than 10 articles. The author with the highest number of publications is Ira Tabas from Columbia University, with a total of 35 articles and 11,329 citations. The second highest is Geoff H Werstuck from McMaster University, with 22 articles and 1,356 citations. Richard C Austin from Henderson Research Center ranks third with 17 articles and 1,582 citations. Yuanyuan Shi from McMaster University is the fourth with 13 articles and 386 citations. Lastly, Ebru Erbay from Ihsan Dogramaci Bilkent University has published 11 articles with 1,454 citations.

**TABLE 2:** Top 10 authors in T cell and AS.

Rank	Author	Record Count	% of 1,035	Citations	Average Per Item	H-index	Affiliations	Total link strength
1	Tabas, Ira	35	3.38	11,329	323.69	32	Columbia University	68
2	Werstuck, Geoff H	22	2.13	1,356	61.64	16	McMaster University	64
3	Austin, Richard C	17	1.64	1,582	93.06	15	Henderson Res Ctr	94
4	Shi, Yuanyuan	13	1.26	386	29.69	10	McMaster University	30
5	Erbay, Ebru	11	1.06	1,454	132.18	8	Ihsan Dogramaci Bilkent University	81
6	Zou, Ming-Hui	9	0.87	929	103.22	8	Georgia State University	30
7	Bernal-mizrachi, Carlos	8	0.77	872	109	8	Washington University	47
8	Davies, Peter F	8	0.77	759	94.88	8	University of Pennsylvania	16
9	Passarelli, Marisa	8	0.77	146	18.25	8	Universidade de Sao Paulo	37
10	Tall, Alan R	8	0.77	1,046	130.75	8	Columbia University	34

**FIGURE 5:**
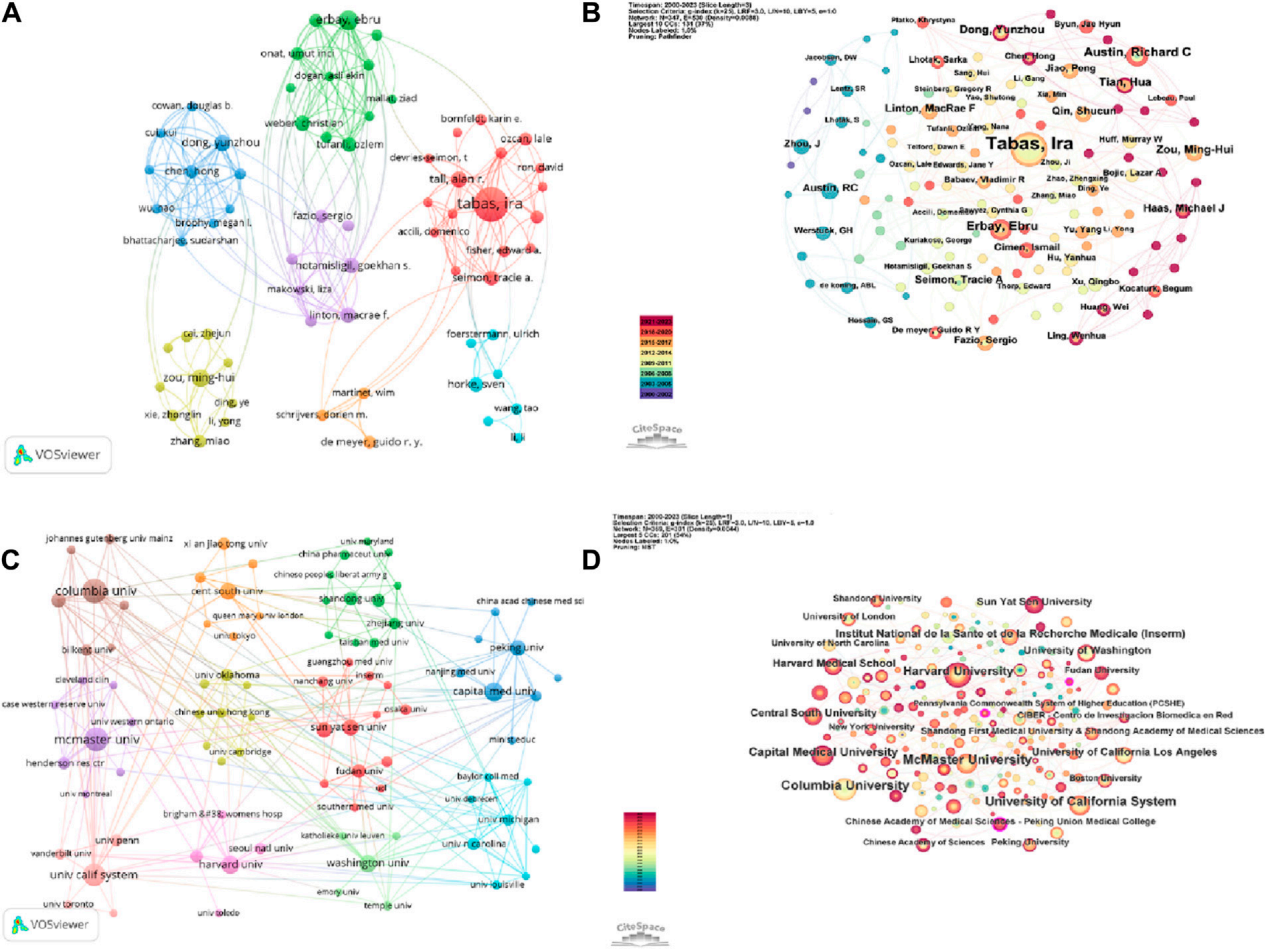
A collaborative network display between authors and institutions from ERS and AS. **(A)** A visual map for the VOSviewer network among authors. **(B)** A visual map for the CiteSpace network among authors. **(C)** A visual map for the VOSviewer network among institutions. **(D)** A visual map for the CiteSpace network among institutions.

VOSviewer parameters were set as follows: Methods (Linlog/modularity) and a minimum number of documents of an author: 3. The obtained results were retrieved for 5,361 authors, and 240 met the thresholds. Based on the analysis of collaborative authors, VOSviewer categorizes authors into 7 clusters. To enhance the visual display, the author co-occurrence network is optimized and presented using Pjake software (Figure 5A). Each node in Figure 5A represents an individual author, where the size of the circle corresponds to the number of publications by each author. The lines between nodes depict the collaborative relationships between authors, with thicker lines indicating stronger collaboration. From the figure, it can be observed that Tabas, Ira has close collaborations with authors such as Tall, Alan R, Seimon, Tracie A, and Fisher, Edward A. Similarly, Erbay, Ebru, Weber, Christian, Mallat, Ziad, and other authors have tight collaborations. Dong, yunzhou, Chen, hong, Cowan, Douglas Bdeng, as well as Zou, Minhui, Xie, Zhonglin, Zhang, Miao, and others also exhibit strong collaborative relationships. Figure 5A clearly demonstrates the clustering of the seven authors, with dense connections within each cluster and relatively sparse and thinner connections between different clusters. This suggests that authors have formed stable collaborative networks, with fewer authors breaking existing collaborations to seek new partners. CiteSpace parameters were set as follows: time slice (2000–2023), year per slice (Benjamin et al., 2017), term source(entire selection), node type(author), and selection criteria (top N = 50). Other parameters were left at the default settings. Figure 4B shows the visual map of authors for CiteSpace network. The size of nodes represents the number of outputs of authors, and different colors represent different years. From 2000 to 2023, the color changes from purple to red.

Based on the data in Table 2 and the author’s co-occurrence maps presented by VOSviewer and CiteSpace, it is evident that Tabas, Ira is an absolute core research force in the field of ERS and AS. Authors such as Werstuck, Geoff H and Austin, Richard C have also made significant contributions to research in this field.

As shown in Figure 5 and Table 3, Columbia University is the institution with the highest number of publications (Battson et al., 2017), followed by McMaster University (Milutinović et al., 2020), University of California System (Esse et al., 2019), Harvard University (Ji et al., 2021), and Capital Medical University (Tabas and Ron, 2011). The top five institutions in terms of citation count are Columbia University (13,154), New York University (3,751), University of California System (3,208), University of Washington (3,205), and Harvard University (2,951).

**TABLE 3:** Top 5 institutions based on publications (Rank a) and citations (Rank b).

Rank	organization	Documents	Citations	Total link strength	Centrality
1a	Columbia University	42	13,154	27	0.1
2a	McMaster University	39	2,441	25	0.07
3a	University of California System	37	3,208	33	0.07
4a	Harvard University	33	2,951	26	0.09
5a	Capital Medical University	26	511	16	0.03
1b	Columbia University	42	13,154	27	0.1
2b	New York University	15	3,751	23	0.19
3b	University of California System	37	3,208	33	0.07
4b	University of Washington	16	3,205	29	0.03
5b	Harvard University	33	2,951	26	0.09

The VOSviewer was set as follows: the minimum number of institutions was 5. Of the 1,192 organizations, 96 met the thresholds. By utilizing Pjake software, the clustering of institutions was visualized and presented in Figure 5C. The institutions were divided into 11 clusters. The first cluster includes Boston University, Chongqing Medical University, Fudan University, INSERM, Guangzhou Medical University, Nanchang University, and others. The second cluster includes China Pharmaceutical University, Chinese People’s Liberation Army General Hospital, Huazhong University of Science and Technology, Shandong University, Wuhan University, and so on. The third cluster includes Capital Medical University, Chinese Academy of Sciences, China Academy of Chinese Medical Sciences, Nanjing Medical University, and so on. The forth cluster includes the Chinese University of Hong Kong, Dalian Medical University, Harbin Medical University, Monash University and so on. The fifth cluster includes Cleveland Clinic Foundation, McMaster University, University of Iowa, and so on. The sixth cluster includes Baylor College of Medicine, Johns Hopkins University, National Taiwan University, University of Michigan and so on. The seventh cluster includes University of Tokyo, Xian Jiaotong University, King’s College London, Queen Mary University London and so on. The eighth cluster includes Columbia University, New York University, Yale University, and so on. The ninth clusters includes Harvard Medical School, Harvard University, University of Toledo and so on. The 10th cluster includes University of California System, University of Pennsylvania, University of Toronto and so on. The 11th cluster includes University of Washington, Emory University, Temple University, and so on. The CiteSpace parameters were set as follows: time slicing (2000–2023), year per slice(1), term source is all, and other parameters were set to default values. Figure 4D displays the visual map of the results. Nodes representing Columbia University, McMaster University, and the University of California System are relatively larger, indicating significant contributions of these institutions in the ERS and AS fields. In Figure 5C,D, all nodes are interconnected closely, suggesting extensive collaborations among different institutions.

### 3.4 Distribution of journals

**FIGURE 6:**
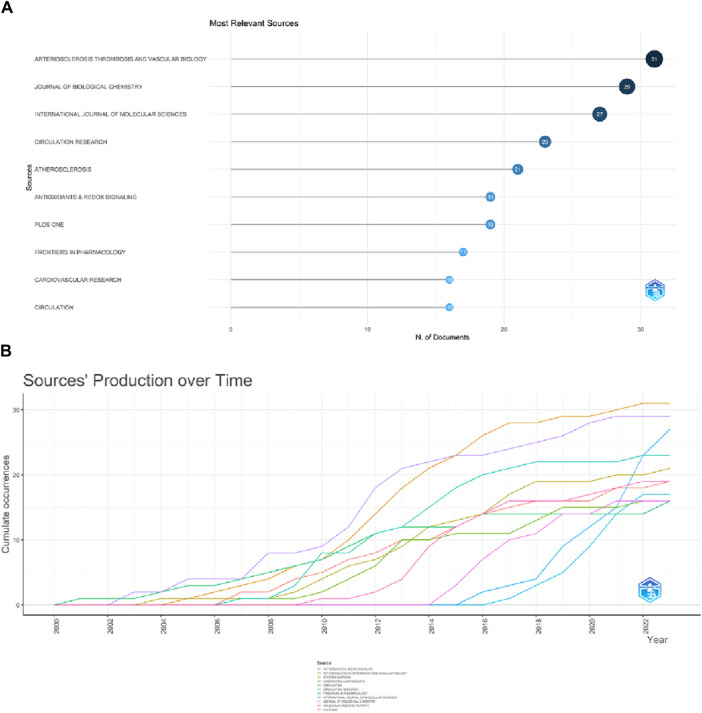
Showcase of issuing journals. **(A)**Top 10 journals in terms of number of publications. **(B)** Trends in the number of articles published in the top ten journals over time.

A total of 1,035 articles were published in 373 journals. Figure 6 and Table 4 present the top ten journals with the highest number of publications, along with their JCR zones and impact factors. Arteriosclerosis Thrombosis and Vascular Biology published the highest number of articles in the ERS and AS fields (31.3%), followed by the Journal of Biological Chemistry (29, 2.8%), International Journal of Molecular Sciences (27, 2.61%), Circulation Research (23, 2.22%), and Atherosclerosis (21, 2.03%). Circulation had the highest IF of 37.8. Among the top 10 journals, 7 (Arteriosclerosis Thrombosis and Vascular Biology, International Journal of Molecular sciences, Circulation Research, Antioxidants & Redox Signaling, Frontiers in Pharmacology, Cardiovascular research and Circulation) journals are all located in the Q1 JCR division, and their IF exceed 5. Figure 6A illustrates the publication volume of the top 10 journals, while Figure 6B displays the temporal trend of publication volume for these journals. It can be observed from the figures that the publication volume of each journal has been increasing over the years, particularly since 2010, when there has been a significant surge in the number of publications.

**TABLE 4:** The top 10 journals in ERS and AS.

Rank	Sources	N(%)	IF^a^(2022)	JCR^b^ (2022)
1	ARTERIOSCLEROSIS THROMBOSIS AND VASCULAR BIOLOGY	31(3.00)	8.700	Q1
2	JOURNAL OF BIOLOGICAL CHEMISTRY	29(2.80)	4.800	Q2
3	INTERNATIONAL JOURNAL OF MOLECULAR SCIENCES	27(2.61)	5.600	Q1
4	CIRCULATION RESEARCH	23(2.22)	20.100	Q1
5	ATHEROSCLEROSIS	21(2.03)	5.300	Q2
6	ANTIOXIDANTS & REDOX SIGNALING	19(1.84)	6.600	Q1
7	PLOS ONE	19(1.84)	3.700	Q2
8	FRONTIERS IN PHARMACOLOGY	17(1.64)	5.600	Q1
9	CARDIOVASCULAR RESEARCH	16(1.55)	10.800	Q1
10	CIRCULATION	16(1.55)	37.800	Q1

^a^ IF: impact factor.

^b^ JCR: journal citation reports.

**FIGURE 7:**
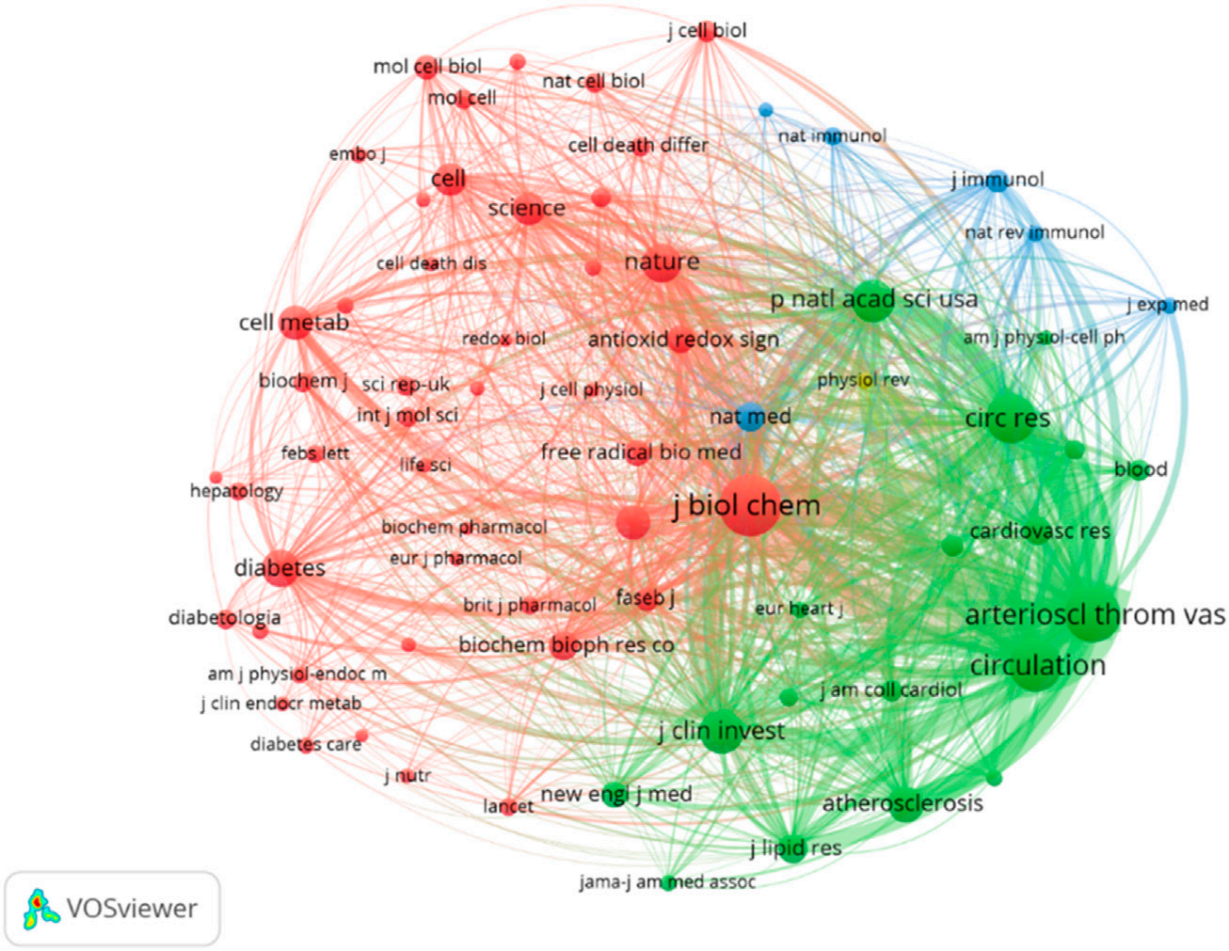
Visualization of co-cited journals. According to the journal content, four clusters—red, green, blue, and yellow—are presented, with the red cluster comprising the most journals and being the largest cluster.

Table 5 presents the top 10 most cited journals, with seven of them ranked in Q1 of JCR. The highest impact factor journal is Nature (64.8), while the top three most cited journals are the Journal of Biological Chemistry (4,011 citations), Arteriosclerosis Thrombosis and Vascular Biology (3,196 citations), and Circulation (2,881 citations), all outstanding journals in JCR zones one and 2. The VOSviewer was set to display a minimum of 200 institutions, with 70 sources meeting the thresholds. Figure 7 shows that the co-citation network of journals is composed of four clusters, corresponding to the four colors in the figure. In the first and second clusters, the research is related to medicine and biochemistry. The first cluster focuses on the microbial level of research, such as cells, while the second cluster is more related to cardiovascular diseases. The third cluster is associated with inflammation and immunity, and the fourth cluster consists of only one review medical journal. The main purpose of citing these clustered journals is to analyze and review existing research, providing theoretical and empirical support for future studies. Among them, the results of the second and third clusters indicate that in the fields of ERS (Environmental and Resources Science) and AS (Aerosol Science), more scholars focus on cardiovascular diseases within the circulatory system. The research hotspot lies in the investigation of inflammatory and immune mechanisms.

**TABLE 5:** Top 10 co-cited journals in T cell and AS.

Rank	Sources	Frequency	IF^a^(2022)	JCR^b^ (2022)
1	Journal Of Biological Chemistry	4,011	4.8	Q2
2	Arteriosclerosis Thrombosis And Vascular Biology	3,196	8.7	Q1
3	Circulation	2,881	37.8	Q1
4	Circulation Research	2,604	20.1	Q1
5	Journal Of Clinical Investigation	2,101	15.9	Q1
6	Proceedings of The National Academy Of Sciences of The United States	1987	11.1	Q1
7	Nature	1,676	64.8	Q1
8	Diabetes	1,509	7.7	Q1
9	Atherosclerosis	1,332	5.3	Q2
10	Plos One	1,221	3.7	Q2

^a^ IF: impact factor.

^b^ JCR: journal citation reports.

### 3.5 Co-cited reference and reference burst

**FIGURE 8:**
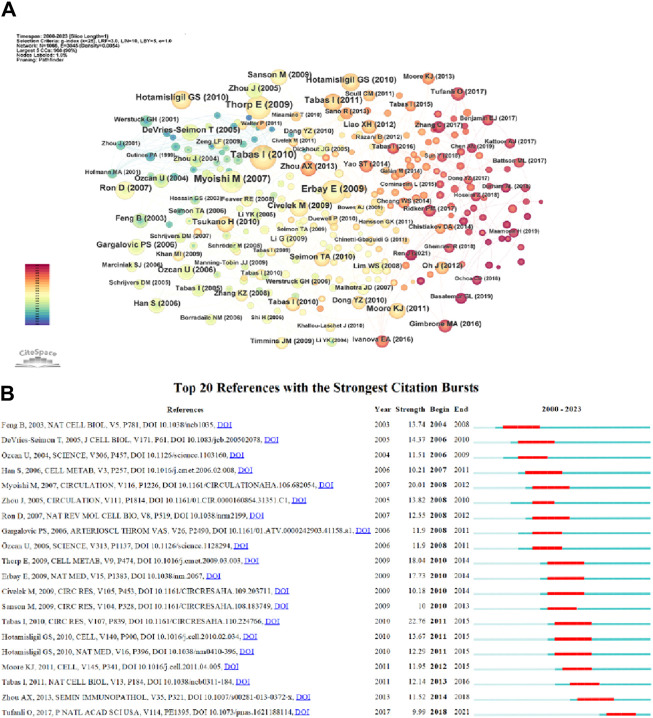
**(A)** References co-citation network in ERS and AS. **(B)** Top 20 references with the strongest citation bursts in ERS and AS.

Co-citation refers to the frequency at which two documents are both cited by another document. The table displays the top ten most cited articles. The most frequently cited article is “The Role of Endoplasmic Reticulum Stress in the Progression of Atherosclerosis” by Ira Tabas, published in Circulation Research (66 citations) (Tabas, 2010). The second one is “Reduced Apoptosis and Plaque Necrosis in Advanced Atherosclerotic Lesions of Apoe/and Ldlr/Mice Lacking CHOP” by Edward Thorp, published in Cell Metabolism (58 citations) (Thorp et al., 2009). The third one includes two articles: “Reducing endoplasmic reticulum stress through a macrophage lipid chaperone alleviates atherosclerosis” by Ebru Erbay, published in Nature Medicine (57 citations) (Erbay et al., 2009), and “Increased Endoplasmic Reticulum Stress in Atherosclerotic Plaques Associated With Acute Coronary Syndrome” by Masafumi Myoishi, MD, published in Coronary Heart Disease (57 citations) (Myoishi et al., 2007). Among the top ten cited articles, five are review articles and five are experimental articles. Based on the co-citation map of literature (Figure 8A), co-citation literature emergence detection was performed, and Figure 8B shows the top 20 most prominent reference literature. The blue line represents the timeline, and the red part on the blue timeline represents the start year, end year, and duration of emergence. The strongest emerging reference literature is “The Role of Endoplasmic Reticulum Stress in the Progression of Atherosclerosis” published in 2010, with an intensity of 22.76. These articles are outstanding contributions in the ERS and AS fields. Among them, “The Role of Endoplasmic Reticulum Stress in the Progression of Atherosclerosis” discusses the role and treatment strategies of endoplasmic reticulum stress in mediating cell apoptosis and inflammatory responses in endothelial cells, smooth muscle cells, and macrophages in atherosclerosis (Tabas, 2010). The high citation and high intensity of emergence both indicate the important influence of this review article.

### 3.6 Research hotspots and frontier analysis

Keywords are important vocabulary that can summarize the main content of a document. The frequency of keywords reflects the changing trends of research in different periods. Visual analysis of keywords provides an intuitive understanding of the research trends in the ERS and AS fields, which helps track the development dynamics of the ERS and AS research areas.

As displayed in Table 6, in addition to endoplasmic reticulum stress(887) and atherosclerosis(586), keywords with higher frequency in this study include apoptosis(254), oxidative stress(248), unfolded protein response(223), inflammation(200), vascular endothelial cell(180), cardiovascular disease(174), activation(160), smooth muscle cells(150). Among these keywords, expression, nf-kappa b, macrophage, insulin resistance, and ldl appeared more than 100 times, indicating that they are the focus of the research.

**TABLE 6:** Top 20 keywords in ERS and AS.

Rank	Keyword	occurrences	Total link strength	Rank	Keyword	occurrence	Total link strength
1	endoplasmic reticulum stress	887	5,593	11	expression	132	884
2	atherosclerosis	586	4,010	12	nf-kappa b	130	917
3	apoptosis	254	1779	13	macrophage	129	914
4	oxidative stress	248	1,682	14	insulin resistance	128	914
5	unfolded protein response	223	1,521	15	ldl	105	720
6	inflammation	200	1,410	16	diabetes mellitus	89	649
7	vascular endothelial cell	180	1,347	17	signaling pathway	83	601
8	cardiovascular disease	174	1,188	18	cholesterol	74	533
9	activation	160	1,153	19	mice	74	512
10	smooth muscle cells	150	1,075	20	gene expression	72	476

**FIGURE 9:**
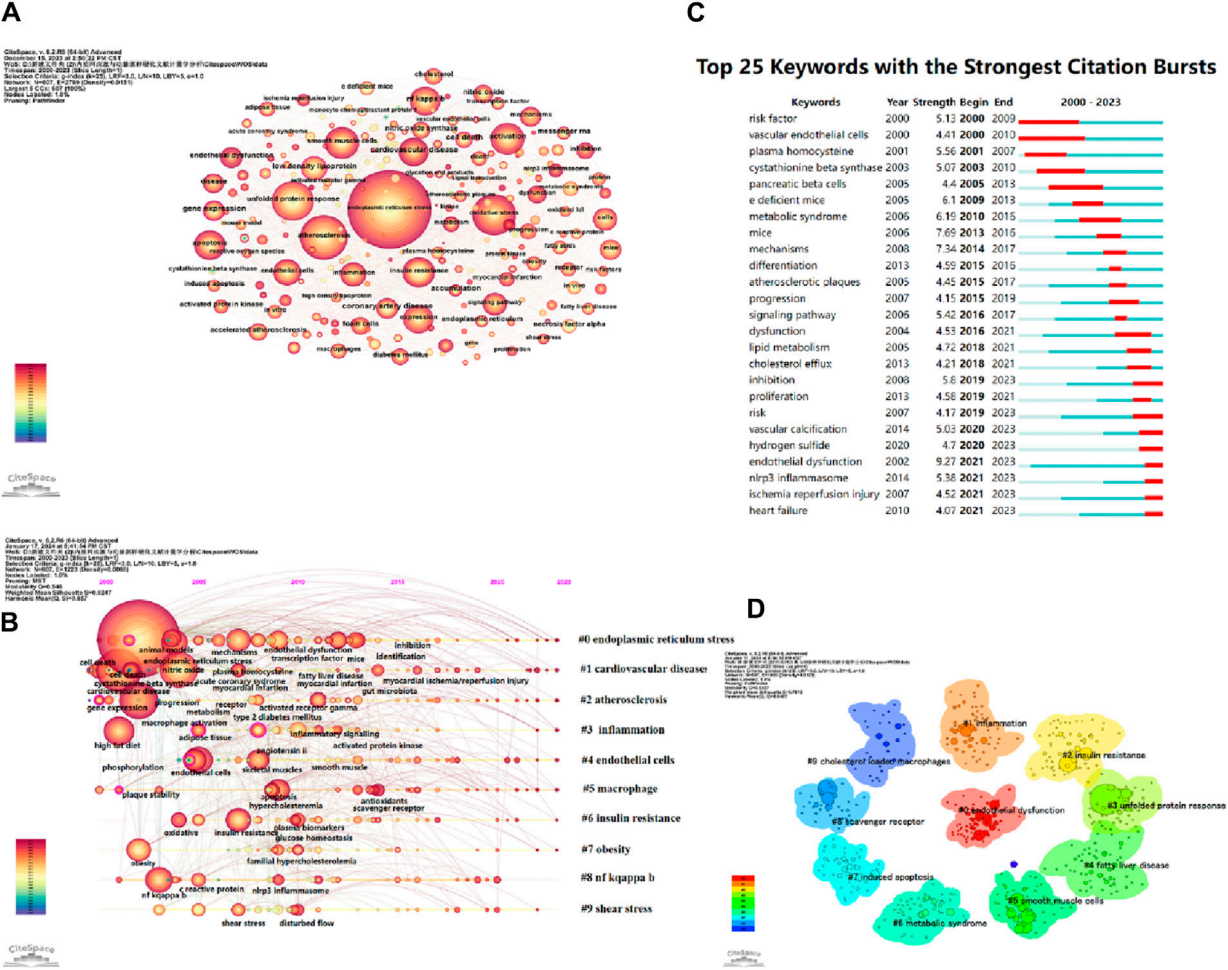
**(A)** Keywords network in ERS and AS. **(B)** Timeline viewer related to ERS and AS. **(C)** Top 25 keywords with the strongest citation bursts. **(D)** The cluster of keywords in the studies of ERS and AS.

CiteSpace parameters were set as follows: time slice (2000–2023), year per slice (Benjamin et al., 2017), term source(entire selection), node type(author), and selection criteria (top N = 50). Other parameters were left at the default settings. The larger the nodes in the graph, the higher the frequency of occurrence of the corresponding keywords. A larger node centrality also indicates the importance of the keyword. The redder the node color, the newer the keyword. Thicker lines between nodes indicate a closer relationship between them. Based on the co-occurrence graph of keywords, we generated keyword clustering graphs, keyword timeline graphs, and the top 25 keywords with the highest prominence intensity (Figure 9).

The timeline graph displays the dynamic evolution path of research hotspots represented by keywords. It explores the evolution process of research hotspots in different periods and helps scholars track current and potential hotspots. Figure 9B visually shows the phased research hotspots and development trends in the ERS and AS fields from a temporal perspective. Multiple clusters are formed, labeled as #0, #1, #2, etc. Figure 9B presents 10 clusters, namely, oxidative stress, cholesterol efflux, insulin resistance, express, endothelial cell, endothelial dysfunction, vitamin D, cell death, unfolded protein response, and cystathionine beta-synthase.

The keyword burst graph intuitively displays the phased changes in research hotspots over time. As shown in Figure 9C, the research hotspots from 2000 to 2010 include vascular endothelial cells, risk factors, nitric oxide, plasma homocysteine, and cystathionine beta-synthase, among others. The hotspots from 2017 to 2023 include cardiovascular disease, lipid metabolism, inhibition, nlrp3 inflammasome, and ischemia reperfusion injury, among others. These keywords are also in the period of research outbreak and their popularity shows no sign of decline over time.

Keyword clustering is a classification based on the degree of association between keywords. In CiteSpace software, the likelihood ratio (LLR) is used to create keyword clustering graphs, where a higher number of clustered nodes indicates a higher research hotspot and a smaller cluster index. The modularity value (Q) and average silhouette value (S) are two metrics used to evaluate the clustering effectiveness of CiteSpace. A Q value greater than 0.3 indicates a significant community structure, while an S value above 0.5 suggests reasonable clustering. When the S value reaches 0.7, it is considered to have high clustering efficiency and is convincing (Yue et al., 2015).

This study formed 10 clusters (Q = 0.5427, S = 0.7818) with convincing clustering results (shown in Table 7). The cluster labels are endothelial dysfunction, inflammation, insulin resistance, unfolded protein response, fatty liver disease, smooth muscle cells, metabolic syndrome, induced apoptosis, scavenger receptor, and cholesterol-loaded macrophages. Clusters #0, #6, and #9 mainly involve the pro-AS effects of ERS on different cells. Clusters #1, #2, #3, #7, and #8 mainly involve the specific mechanism by which ERS promotes AS. Clusters #4 and #6 are related to diseases associated with ERS and AS.

**TABLE 7:** Ten keyword clusters.

Cluster groups	Key words
#0 endothelial dysfunction	endothelial dysfunction, nitric oxide, shear stress, hydrogen sulfide, endothelial growth factor, plasma homocysteine, monocyte chemoattractant protein 1, transcription factor xbp1, vascular remodeling
#1 inflammation	Inflammation, macrophages, TNF-α, T cells, AMPK, nlrp3 inflammasome
#2 insulin resistance	insulin resistance, type 2 diabetes mellitus, lipid metabolism, protein kinase c, oxidative stress
#3 unfolded protein response	unfolded protein response, NF kappa B, c/ebp homologous protein, free cholesterol, macrophage apoptosis, foam cells, vulnerable plaque, bcl-2 genes, ATF4
#4 fatty liver disease	fatty liver disease, high density lipoprotein, diet induced obesity, ubiquitin proteasome system, familial hypercholesterolemia, cholesterol oxidation products
#5 smooth muscle cells	smooth muscle cells, programmed cell death, glutathione peroxidase
#6 metabolic syndrome	metabolic syndrome, ischemia reperfusion injury, alzheimers disease, heart failure, nonalcoholic steatohepatitis
#7 induced apoptosis	induced apoptosis, macrophage polarization, PPAR-γ, transcription factor foxo1, liver x receptor, ldl oxidation, carboxy terminal hydrolase
#8 scavenger receptor	scavenger receptor, adhesion molecules, phagocytosis, necrosis, kinase 3 beta, c reactive protein, palmitic acid
#9 cholesterol loaded macrophages	cholesterol loaded macrophages, lipid peroxidation, 9 oxononanoyl cholesterol, apoptosis susceptibility, serum paraoxonase, biological activity

## 4 Discussion

### 4.1 General information of main findings

This research conducted bibliometric analysis on 1,035 documents from the core collection of the Web of Science database, spanning from 1 January 2000, to 1 December 2023. Utilizing software such as Citespace, Vosviewer, and the R package “bibliometric”, the study aimed to visually analyze publications, countries, institutions, journals, and co-cited literature in the fields of ERS and AS. The findings revealed a consistent increase in literature publications in the ERS and AS over the initial decade from 2000 to 2013, followed by a fluctuating upward trend, indicating sustained scholarly attention to the field. The United States and China emerged as principal research countries, with literature from countries like the United Kingdom, the United States, Germany, and France exhibiting a high citation rate, signifying the quality and maturity of research in the field.

In terms of authors and journals, specific individuals such as Ira Tabas from Columbia University and certain journals like Circulation demonstrated significant impact and importance in the field. These results delineate the trajectory of research development in the ERS and AS, highlighting research hotspots and emerging frontiers. They assist scholars in selecting appropriate publication outlets and collaborators while providing theoretical underpinnings for future investigations in the field.

### 4.2 Knowledge base of ERS and AS

Co-citation refers to two articles being cited by another article at the same time. Highly co-cited research is usually considered the foundation of a field (Lijun et al., 2019). Table 8 shows the top ten highly cited articles, including five reviews and five experimental articles. These ten articles explore the relationship between ERS and AS from different perspectives.

**TABLE 8:** Top 10 cited references of publications in ERS and AS.

Rank	Author	Title	Journal	Co-citation	Centrality
1	Ira Tabas	The Role of Endoplasmic Reticulum Stress in the Progression of Atherosclerosis	Circulation Research	66	0.01
2	Edward Thorp	Reduced Apoptosis and Plaque Necrosis in Advanced Atherosclerotic Lesions of Apoe/and Ldlr/Mice Lacking CHOP	Cell Metabolism	58	0.07
3	Ebru Erbay	Reducing endoplasmic reticulum stress through a macrophage lipid chaperone alleviates atherosclerosis	Nature Medicine	57	0.02
4	Masafumi Myoishi, MD	Increased Endoplasmic Reticulum Stress in Atherosclerotic Plaques Associated With Acute Coronary Syndrome	Coronary Heart Disease	57	0.05
5	Hotamisligil GS	Endoplasmic Reticulum Stress and the Inflammatory Basis of Metabolic Disease	Cell	40	0.02
6	Ira Tabas	Integrating the mechanisms of apoptosis induced by endoplasmic reticulum stress	Review	37	0.15
7	David Ron	Signal integration in the endoplasmic reticulum unfolded protein response	REVIEWS	36	0.03
8	Hotamisligil GS	Endoplasmic reticulum stress and atherosclerosis	COMMENTARY	36	0
9	Mete Civelek	Chronic Endoplasmic Reticulum Stress Activates Unfolded Protein Response in Arterial Endothelium in Regions of Susceptibility to Atherosclerosis	Circulation Research	33	0.02
10	Tracie DeVries-Seimon	Cholesterol-induced macrophage apoptosis requires ER stress pathways and engagement of the type A scavenger receptor	The Journal Of Cell Biology	31	0.16

A review published by Ira Tabas in the journal Circulation in 2010 systematically discusses the role of endoplasmic reticulum stress (ERS) in the development of atherosclerosis. This article summarizes that under stimuli such as oxidative stress, oxidized sterols, elevated intracellular cholesterol levels, and saturated fatty acids, the unfolded protein response (UPR) can be prolonged and activated. It provides ample evidence to demonstrate the chronic activation of UPR in atherosclerotic lesion cells, such as endothelial cells, smooth muscle cells, and macrophages. The long-term activation of ERS is believed to influence the formation and vulnerability of atherosclerotic plaques by recruiting inflammatory factors, inducing inflammatory responses, and activating multiple pro-apoptotic signaling pathways (Tabas, 2010).

The team led by Edward Thorp discovered through experiments that CHOP expression increases in advanced atherosclerotic lesions. By comparing the size and morphology of aortic root lesions in fat-fed Chop+/+; Apoe−/− and Chop−/−; Apoe−/− mice, they found that plaques and apoptotic cells were reduced in CHOP-deficient mice. They suggest that the CHOP pathway may serve as a potential therapeutic target for treating atherosclerosis (Thorp et al., 2009).

Ebru Erbay and colleagues discovered through genetic and chemical models that adipocyte fatty acid-binding protein 4 (aP2) is a major regulatory factor for lipid-induced endoplasmic reticulum stress in macrophages. They found that upregulating the chemical chaperone 4-phenyl butyric acid (PBA) or inhibiting the lipid chaperone aP2 significantly suppressed endoplasmic reticulum stress, cell death, and atherosclerosis in macrophages (Erbay et al., 2009).

Masafumi Myoishi, MD and colleagues detected the expression of endoplasmic reticulum (ER) chaperones and apoptotic cells in smooth muscle cells and macrophages within thin-cap fibroatheromas and ruptured plaques in coronary artery segments obtained during autopsies of 71 patients. They found a significant increase in ER chaperone expression and apoptotic cells. Oxysterols, such as 7-ketocholesterol (7-KC), contribute to the cytotoxicity of oxidized low-density lipoprotein. The upregulation of 7-KC expression induces ER chaperone upregulation through the generation of reactive oxygen species, leading to increased endoplasmic reticulum stress and activation of the downstream CHOP signaling pathway, which induces apoptosis in smooth muscle cells and macrophages. Further experiments revealed the activation of the Chop-dependent pathway in unstable plaques. Therefore, endoplasmic reticulum stress is considered one of the factors that increase vulnerability of coronary artery plaques, leading to acute coronary syndrome and fatal outcomes in patients with coronary artery disease (Myoishi et al., 2007).

Gökhans S. Hotamisligil reviewed the cross-talk between the endoplasmic reticulum (ER) stress response and inflammatory pathways in the context of metabolic homeostasis and disease, highlighting the importance of ER stress as a crucial mechanism underlying metabolic diseases. The author proposed that ER stress-induced inflammation and autophagy are also significant causes of metabolic dysfunction. By summarizing these viewpoints, the author suggested that there is enormous potential for targeting ER stress in the treatment of metabolic diseases (Hotamisligil, 2010a).

Ira Tabas provided a comprehensive review on the molecular mechanisms of endoplasmic reticulum (ER) stress and cell apoptosis, focusing on the pathophysiological relationship between ER stress and cell apoptosis, as well as the integration and complementarity of various cell apoptosis pathways induced by ER stress. The aim was to provide a basis for future drug intervention strategies (Tabas and Ron, 2011). David Ron and colleagues systematically discussed the three branches of the unfolded protein response (UPR) under ER stress and explained the role of different gene expressions in the secretion pathway. The goal was to preserve the beneficial effects of ER stress in the human body while alleviating its detrimental effects (Ron and Walter, 2007).

Gökhan S. Hotamisligil highlighted the role of endoplasmic reticulum (ER) stress in macrophages exposed to lipotoxic environments, which can activate inflammatory and apoptotic pathways, promoting the formation of atheromatous plaques or accelerating the rupture of vascular plaques and exacerbating metabolic dysfunction. The author proposed that inhibiting or genetically deleting lipid chaperone aP2 could be an important means of enhancing ER folding capacity to alleviate ER stress (Hotamisligil, 2010b). Mete Civelek and colleagues found through experiments that chronic ER stress and UPR response in endothelial cells were generally present in susceptible sites of atherosclerosis *in vivo*. Further research showed that downstream signaling pathways IRE1 and ATF6, as well as their downstream effectors, were activated by the UPR pathway induced by ER stress. Increasing protein folding capacity to alleviate ER stress may be a feasible method for treating atherosclerosis (Civelek et al., 2009). Tracie DeVries-Seimon and colleagues found through experiments that free cholesterol loading in macrophages could induce ER stress, activate p38-CHOP and JNK2 pathways, and induce cell apoptosis, which required the involvement of type A scavenger receptor (SRA) (Devries-Seimon et al., 2005).

The top ten highly cited publications reflect the high academic standards in the field of research on ERS and AS, serving as a crucial knowledge repository. These publications summarize both basic and clinical research findings, providing evidence and guidance for future investigations. Seven of them focused on the pathological mechanisms by which ERS promotes the progression of AS, while three focused on the potential of targeting ERS in treating AS, indicating that the pathological mechanisms of ERS and AS are the primary research issues.

### 4.3 Identification of research hotspots and emerging topics

In bibliometrics, keyword co-occurrence analysis reflects the research hotspots and development trends in the field. A timeline visualization shows the trend of research hotspots over time, while keyword burst analysis helps to track research frontiers. Keyword clustering analysis displays research modules and knowledge structures.

From the keyword co-occurrence map (Figure 9A) and Table 6, it can be observed that keywords such as apoptosis, oxidative stress, unfolded protein response, inflammation, vascular endothelial cell, smooth muscle cells, NF-κB, macrophage, etc. represent the main research directions and have constructed the knowledge structure of this field. The appearance of vascular endothelial cells, risk factors, plasma homocysteine, cystathionine beta synthase, and pancreatic beta cells from 2000 to 2005 indicated the initial focus of research. The subsequent appearance of keywords such as e deficient mice, metabolic syndrome, mice, mechanisms, differentiation, signaling pathway, cholesterol efflux, etc. enriched the knowledge base of ERS and AS fields. With the continuous improvement and innovation of experimental techniques, numerous scholars have conducted more in-depth and diversified research on the cellular and molecular mechanisms in the ERS and AS fields. In recent 5 years, related studies such as vascular calcification, endothelial dysfunction, NLRP3 inflammation, ischemia-reperfusion injury, and heart failure have become new hotspots, and are currently in a burst phase.

Through keyword clustering analysis, we can gain a more intuitive understanding of the main directions in the field of ERS and AS over the past 20 years. The figure displays a total of 10 clusters, and based on the content of keywords within each cluster, we categorized them into three main research directions.

#### 4.3.1 The pro-atherosclerotic effects of ER stress on different cells

Endothelial cells, macrophages, and smooth muscle cells are all related to the occurrence and development of atherosclerosis. The dysfunction of endothelial cells, polarization of macrophages, and apoptosis and migration of smooth muscle cells all contribute to the occurrence and progression of atherosclerosis. Vascular endothelial cells (VECs) are the barrier between the vascular wall and blood circulation and are important cells for maintaining the stability of the vascular wall (Krüger-Genge et al., 2019). Endothelial dysfunction is the initiating factor of atherosclerosis. Atherosclerosis often occurs in areas with turbulent blood flow, where endothelial cells are subjected to continuous blood flow strain. Unstable blood flow shear stress is a significant contributor to endothelial dysfunction, capable of directly inducing AS. (Chung et al., 2015). Oxidized low-density lipoprotein and homocysteine are independent risk factors for atherosclerosis and can directly induce ERS in VECs (Lubrano and Balzan, 2014; Ji et al., 2021). Excessive ERS will activate pro-apoptotic pathways and recruit inflammatory factors. HANG et al. found that in ox-LDL-induced human aortic endothelial cells (HAECs), ERS-related proteins such as CHOP, p-PERK, GRP78, NLRP3, and interleukin-1β were upregulated, suggesting that ERS induces VECs apoptosis and mediates inflammation to promote endothelial dysfunction (Hang et al., 2020). Asymmetric dimethylarginine (ADMA) is an endogenous inhibitor of nitric oxide synthase (NOS). It competes with L-arginine for binding to the active site of NOS, reducing the production of nitric oxide (NO) and promoting the generation of reactive oxygen species (ROS). ADMA has been shown to be closely associated with atherosclerosis (AS) (Lee et al., 2021). Interestingly, in the experiment conducted by Toyomasu et al., it was found that levels of homocysteine (Hcy) and ADMA were highly correlated (Toyomasu et al., 2021). Elevated levels of Hcy can increase ADMA levels through the induction of oxidative stress (Esse et al., 2019), which in turn stimulates endothelial cell endoplasmic reticulum stress (ERS) and leads to endothelial dysfunction. Activated and dysfunctional endothelial cells promote the upregulation of adhesion molecules and chemokines, as well as the secretion of vascular endothelial growth factor, resulting in the formation of lipid plaques and vascular remodeling. Additionally, the inflammatory state of endothelial cells exacerbates plaque instability (Milutinović et al., 2020; Björkegren and Lusis, 2022). Increasing evidence suggests that sustained ERS caused by various factors leading to changes in the intracellular and extracellular environment of endothelial cells is an important mechanism underlying endothelial dysfunction. The role of ERS-induced unfolded protein response (UPR) in promoting apoptosis and activating inflammatory responses in endothelial dysfunction has drawn the attention of many researchers.

Macrophages are the main effector cells in the occurrence and progression of AS (Moore and Tabas, 2011). Studies have also shown that ERS-induced macrophage apoptosis is a key factor in plaque vulnerability and rupture (Myoishi et al., 2007). Numerous experimental studies have demonstrated that ERS-related protein expression is upregulated in macrophages from rat models at all stages of AS (Devries-Seimon et al., 2005; Zhou et al., 2005), with UPR-mediated activation of CHOP and downstream apoptotic signaling pathways being one of the most common mechanisms underlying ERS-induced macrophage apoptosis (Battson et al., 2017). Wu et al. (Wu et al., 2018) found that ox-LDL induces macrophage secretion of high mobility group B-1 (HMGB-1) through oxidative stress, and HMGB-1 promotes macrophage apoptosis and foam cell formation by activating the CHOP pathway. In addition, ox-LDL can induce upregulation of scavenger receptors CD36 and SR-A through ERS induction (Yao S. et al., 2014; Choromańska et al., 2017; Kattoor et al., 2019). Meanwhile, activation of UPR signaling pathways downregulates expression of ABCA1, ABCG1, and SR-BI, reducing cholesterol efflux, affecting macrophage lipid metabolism, and promoting macrophage phenotypic transformation into foam cells. Foam cell formation is the most important pathological hallmark of the entire AS process (Yao ST. et al., 2014). Yao et al. (Yao ST. et al., 2014) found that minimally modified low-density lipoprotein (mm-LDL) induces the accumulation of free cholesterol (FC) in the endoplasmic reticulum (ER), stimulating macrophage ERS and activating the ATF6 and p-IRE1-mediated UPR signaling pathway through Toll-like receptor 4 (TLR4). This leads to activation of JNK and p38-CHOP-Bax-mediated apoptosis, promoting cell death and plaque instability. In addition, research results have also indicated a close association between ERS and the regulation of macrophage polarization in inflammatory diseases such as atherosclerosis, tumors, and pulmonary fibrosis (Oh et al., 2012; Cook et al., 2016). Studies have shown that all three pathways of UPR activated by ERS can be associated with M1 polarization of macrophages through downstream factors such as JNK-AP1 and NF-κB, regulating macrophage polarization towards M1 and inducing inflammatory responses (Ke et al., 2017). Through the study of macrophage subtypes in plaques, CHO found that M2-type macrophages are highly distributed in stable plaques, while M1-type macrophages are predominant in unstable plaques, especially in the shoulder region of the intima prone to rupture (Cho et al., 2013). The above research results suggest that ERS induces macrophage polarization towards M1, resulting in high expression of M1-type macrophages and promoting plaque rupture.

ERS-induced apoptosis of vascular smooth muscle cells (VSMCs) leads to thinning of the protective fibrous cap and may be one of the important mechanisms contributing to the transition of atherosclerotic plaques from stable to unstable (Yao et al., 2012; Liu et al., 2016). Protein kinase C (PKC) is a key regulatory factor in VSMC apoptosis and can participate in ERS-dependent apoptotic signaling through the IRE1α/JNK pathway (Larroque-Cardoso et al., 2013). Vascular calcification is a prominent feature of atherosclerosis, and experimental evidence suggests that elevated expression of GRP78 in calcified arteries indicates that ERS promotes the phenotypic transformation of VSMCs into osteoblast-like cells, leading to vascular calcification (Duan et al., 2009). In addition, hyperhomocysteinemia (HHcy) can also significantly activate the arm of endoplasmic reticulum protein (HERP) in VSMCs, inducing VSMC phenotype transformation (Lin et al., 2018).

The mechanisms by which ERS promotes atherosclerosis in different cell types are complex and involve various physiological and pathological responses, including apoptosis, inflammatory reactions, oxidative stress, and disturbances in lipid metabolism. Therefore, ERS is also a hot and focal point of research.

#### 4.3.2 Mechanisms underlying the promotion of AS by ERS

It is known that the unfolded protein response (UPR) is an adaptive response to perturbations in endoplasmic reticulum (ER) homeostasis caused by various pathological factors. The primary goal of the UPR is to adjust and restore ER function through inhibition of translation, upregulation of ER chaperones, and degradation of unfolded proteins (Engin and Hotamisligil, 2010). Persistent ERS can lead to the activation of apoptotic and inflammatory pathways. C/EBP homologous protein (CHOP), a widely studied biomarker of ERS-related apoptotic signaling pathways, induces apoptosis by upregulating the expression of members of the Bcl-2 family to activate the apoptotic signaling pathway mediated by CHOP. In addition, the activated IRE1 binds to TNF receptor-associated factor 2 (TRAF2) to form a complex that activates JNK and p38 MAPK via the apoptotic signal-regulating kinase 1 (ASK1), which also induces apoptosis (Hong et al., 2017). At the same time, ERS activates inflammatory pathways through various signaling pathways, activating the NLRP3 inflammasome and recruiting large amounts of inflammatory factors such as IL-8, IL-6, monocyte chemoattractant protein-1 (MCP-1), and tumor necrosis factor-α (TNF-α), triggering inflammatory responses (Li et al., 2005). ERS-mediated apoptosis and inflammatory responses are widely involved in all stages of atherosclerosis.

Insulin resistance (IR) is recognized as a key pathogenic factor and a major cause of various metabolic abnormalities associated with cardiovascular diseases (Yan, 2005). IR can mediate changes in C-peptide and adiponectin levels, leading to dysregulation of lipid metabolism and promoting the development of atherosclerosis (Linna and Lifeng, 2017). Due to impaired pancreatic function accompanying IR, the body enters a state of hyperglycemia. This activates signaling pathways such as polyols, hexosamines, advanced glycation end products, and protein kinase C, enhancing the activity of reduced nicotinamide adenine dinucleotide phosphate oxidase, resulting in the generation of a large number of reactive oxygen species (ROS). This leads to increased oxidative stress levels, affects endoplasmic reticulum (ER) homeostasis, activates ER stress (ERS) (Lindholm et al., 2017), and mediates high expression of inflammatory factors such as NF-κB, TNF-α, matrix metalloproteinases, causing endothelial dysfunction, collagen fiber proliferation, and smooth muscle cell apoptosis. Interestingly, under the influence of IR, macrophages become more sensitive to ER stress, activating inflammatory responses and apoptotic signaling pathways, thereby promoting the progression of atherosclerosis (Schmitz et al., 2018).

Numerous experimental studies have found that endoplasmic reticulum stress (ERS) can promote macrophage apoptosis in advanced stage atherosclerotic (AS) lesions, and it has been discovered that ERS-induced macrophage apoptosis requires the involvement of scavenger receptor A (SRA) (Feng et al., 2003a; Tabas, 2005; Lim et al., 2008). Scavenger receptors are mainly expressed on the surface of macrophages in different tissues and organs and mediate lipid internalization by recognizing changes in the surface molecular patterns of chemically modified low-density lipoprotein, thereby forming foam cells (Suzuki et al., 1997). Macrophages at the early stage of arterial atherosclerotic lesion damage have normal cholesterol transport capabilities. Cholesterol taken up by the surface scavenger receptor can be esterified into fatty acids in the endoplasmic reticulum through acyl-CoA: cholesterol acyltransferase (ACAT) and transferred out of the cell membrane or to the extracellular space (Tabas, 2000). However, in late-stage injury, both ACAT activity and cholesterol efflux function of macrophages are impaired, leading to the accumulation of a large amount of free cholesterol (FC) (Shio et al., 1979; Small et al., 1984). The accumulation of FC can activate the unfolded protein response (UPR) and its downstream apoptotic signaling pathways, causing macrophage apoptosis (Feng et al., 2003a; Feng et al., 2003b).

#### 4.3.3 Diseases associated with ERS and AS

AS is a significant pathological basis for vascular diseases worldwide. The progression of AS leads to gradual narrowing of the lumen, resulting in blood flow obstruction, as well as unstable plaque rupture leading to acute thrombosis and subsequent ischemic necrosis of affected organs, which is a major cause of sudden death. Its main clinical manifestations include ischemic heart disease (IHD), ischemic stroke, and peripheral arterial disease (Herrington et al., 2016). Additionally, the incidence of AS increases with age. Furthermore, ERS plays a role in diseases such as non-alcoholic fatty liver disease (NAFLD) and Alzheimer’s disease and is closely associated with the development of AS. As a metabolic disease, NAFLD is closely related to oxidative stress and ERS (Ashraf and Sheikh, 2015). ERS activates the PERK-eIF2α pathway, regulates lipid synthesis and degeneration, increases *de novo* lipogenesis, and promotes lipid deposition in liver cells (Tian et al., 2018). The severity of hepatic steatosis can predict the risk of future cardiovascular events (Pisto et al., 2014). Multiple studies have reported that NAFLD patients have a significantly higher risk of developing coronary heart disease, and the most common cause of death in NAFLD patients is cardiovascular disease (CVD) (Targher et al., 2016; Golabi et al., 2019). The pathological mechanism of AD involves abnormal extracellular deposition of β-amyloid protein in the brain, leading to the destruction of surrounding neurons and synapses (Nebel et al., 2018). It has been discovered that the abnormal accumulation of Aβ induces ERS and activates the UPR and its downstream signaling pathways, upregulating NF-κB to activate glial cells, induce neuroinflammation, and exacerbate the progression of AD (Salminen et al., 2020). On the other hand, AS can promote the accumulation of β-amyloid protein in the brain by reducing cerebral blood flow and causing cerebral hypoxia (Matthews et al., 2019; Takousis et al., 2019). In turn, Aβ promotes the progression of AS through endothelial dysfunction, oxidative stress, and inflammatory responses (Gupta and Iadecola, 2015; Liu et al., 2019), forming a vicious cycle. Research on diseases related to ERS and AS is also a hot topic of study.

Our findings reveal that the mechanistic study of ERS and AS has been a hot topic of research, and the enthusiasm of scholars in the study of related diseases has continued to rise with the passage of time. Although significant progress has been made in ERS and AS related research, it is found through our study that further research is still needed in this field to fill some knowledge gaps: a. The mechanisms related to ERS-induced inflammatory response and activation of apoptotic pathway to promote the progression of AS are relatively well established, while there are relatively few data related to biological targets that modulate or restore the function of ER. b. As shown by the results of the present study, the vast majority of the highly co-cited literature are basic research or review articles, while influential clinical trials and practice guidelines are quite lacking, suggesting that the research results in this field have not fully realized the widespread application from laboratory to clinic. Meanwhile, through the trend of research hotspots over time and the gaps found in this study, we can speculate the future research trends in this field: a. Research on the microscopic mechanism of ERS and AS. b. Screening of biological targets to modulate the function of ER or inhibit ERS and determination of their clinical effects. c. The treatment of diseases related to ERS and AS, as well as the development of new drugs targeting corresponding targets.

## 5 Conclusion

This study utilized CiteSpace, VOSviewer, and the R package of “bibliometric” to perform a visualized analysis of the literature in the field of ERS and AS, providing an intuitive display of the current research status and frontier hotspots in this field. By collecting relevant literature published between 2000 and 2023, we established a knowledge base of ERS and AS and found that ERS has significant research value in understanding the mechanisms and treatments of AS. Analysis of the collaboration network map revealed that China and the United States are the main countries conducting research on ERS and AS, and Ira Tabas is an eminent figure in this field, with highly influential and academically valuable publications. The authors have formed relatively stable collaborative relationships, but breaking through geographical limitations and strengthening further cooperation is still necessary. Scholars are more focused on studying the pathological mechanisms of ERS in promoting the occurrence and development of AS, as well as related diseases and biomarkers, which will be the key areas of future research attention. While paying attention to basic research, researchers should also emphasize the integration with clinical aspects to provide scientific evidence for the treatment of clinically relevant AS-related diseases.

## References

[B1] Adolph T. E. Niederreiter L. Blumberg R. S. Kaser A. (2012). Endoplasmic reticulum stress and inflammation. Dig. Dis. 30 (4), 341–346. 10.1159/000338121 22796794 PMC3423328

[B2] Aria M. Cuccurullo C. Egghe L. (2017). bibliometrix: an R-tool for comprehensive science mapping analysis.

[B3] Ashraf N. U. Sheikh T. A. (2015). Endoplasmic reticulum stress and Oxidative stress in the pathogenesis of Non-alcoholic fatty liver disease. Free Radic. Res. 49 (12), 1405–1418. 10.3109/10715762.2015.1078461 26223319

[B4] Battson M. L. Lee D. M. Gentile C. L. (2017). Endoplasmic reticulum stress and the development of endothelial dysfunction. Am. J. Physiol. Heart Circ. Physiol. 312 (3), H355–H367. 10.1152/ajpheart.00437.2016 27923788

[B5] Benjamin E. J. Blaha M. J. Chiuve S. E. Cushman M. Das S. R. Deo R. (2017). Heart disease and stroke statistics-2017 update: a report from the American heart association. Circulation 135 (10), e146–e603. 10.1161/cir.0000000000000485 28122885 PMC5408160

[B6] Björkegren J. L. M. Lusis A. J. (2022). Atherosclerosis: recent developments. Cell 185 (10), 1630–1645. 10.1016/j.cell.2022.04.004 35504280 PMC9119695

[B7] Bravo R. Vicencio J. M. Parra V. Troncoso R. Munoz J. P. Bui M. (2011). Increased ER-mitochondrial coupling promotes mitochondrial respiration and bioenergetics during early phases of ER stress. J. Cell Sci. 124 (Pt 13), 2143–2152. 10.1242/jcs.080762 21628424 PMC3113668

[B8] Chen C. (2006). CiteSpace II: detecting and visualizing emerging trends and transient patterns in scientific literature. J. Am. Soc. Inf. Sci. Technol. 57 (3), 359–377. 10.1002/asi.20317

[B9] Cho K. Y. Miyoshi H. Kuroda S. Yasuda H. Kamiyama K. Nakagawara J. (2013). The phenotype of infiltrating macrophages influences arteriosclerotic plaque vulnerability in the carotid artery. J. Stroke Cerebrovasc. Dis. 22 (7), 910–918. 10.1016/j.jstrokecerebrovasdis.2012.11.020 23273713

[B10] Choromańska B. Myśliwiec P. Choromańska K. Dadan J. Chabowski A. (2017). The role of CD36 receptor in the pathogenesis of atherosclerosis. Adv. Clin. Exp. Med. 26 (4), 717–722. 10.17219/acem/62325 28691408

[B11] Chung J. Kim K. H. Lee S. C. An S. H. Kwon K. (2015). Ursodeoxycholic acid (UDCA) exerts anti-atherogenic effects by inhibiting endoplasmic reticulum (ER) stress induced by disturbed flow. Mol. Cells 38 (10), 851–858. 10.14348/molcells.2015.0094 26442866 PMC4625066

[B12] Civelek M. Manduchi E. Riley R. J. Stoeckert C. J. Davies P. F. (2009). Chronic endoplasmic reticulum stress activates unfolded protein response in arterial endothelium in regions of susceptibility to atherosclerosis. Circ. Res. 105 (5), 453–461. 10.1161/CIRCRESAHA.109.203711 19661457 PMC2746924

[B13] Cook K. L. Soto-Pantoja D. R. Clarke P. A. Cruz M. I. Zwart A. Wärri A. (2016). Endoplasmic reticulum stress protein GRP78 modulates lipid metabolism to control drug sensitivity and antitumor immunity in breast cancer. Cancer Res. 76 (19), 5657–5670. 10.1158/0008-5472.CAN-15-2616 27698188 PMC5117832

[B14] Devries-Seimon T. Li Y. Yao P. M. Stone E. Wang Y. Davis R. J. (2005). Cholesterol-induced macrophage apoptosis requires ER stress pathways and engagement of the type A scavenger receptor. J. Cell Biol. 171 (1), 61–73. 10.1083/jcb.200502078 16203857 PMC2171235

[B15] Duan X. Zhou Y. Teng X. Tang C. Qi Y. (2009). Endoplasmic reticulum stress-mediated apoptosis is activated in vascular calcification. Biochem. Biophys. Res. Commun. 387 (4), 694–699. 10.1016/j.bbrc.2009.07.085 19622343

[B16] Ellegaard O. Wallin J. A. (2015). The bibliometric analysis of scholarly production: how great is the impact? Scientometrics 105 (3), 1809–1831. 10.1007/s11192-015-1645-z 26594073 PMC4643120

[B17] Engin F. Hotamisligil G. S. (2010). Restoring endoplasmic reticulum function by chemical chaperones: an emerging therapeutic approach for metabolic diseases. Diabetes Obes. Metab. 12 (Suppl. 2), 108–115. 10.1111/j.1463-1326.2010.01282.x 21029307

[B18] Erbay E. Babaev V. R. Mayers J. R. Makowski L. Charles K. N. Snitow M. E. (2009). Reducing endoplasmic reticulum stress through a macrophage lipid chaperone alleviates atherosclerosis. Nat. Med. 15 (12), 1383–1391. 10.1038/nm.2067 19966778 PMC2790330

[B19] Esse R. Barroso M. Tavares de Almeida I. Castro R. (2019). The contribution of homocysteine metabolism disruption to endothelial dysfunction: state-of-the-art. Int. J. Mol. Sci. 20 (4), 867. 10.3390/ijms20040867 30781581 PMC6412520

[B20] Feng B. Yao P. M. Li Y. Devlin C. M. Zhang D. Harding H. P. (2003a). The endoplasmic reticulum is the site of cholesterol-induced cytotoxicity in macrophages. Nat. Cell Biol. 5 (9), 781–792. 10.1038/ncb1035 12907943

[B21] Feng B. Zhang D. Kuriakose G. Devlin C. M. Kockx M. Tabas I. (2003b). Niemann-Pick C heterozygosity confers resistance to lesional necrosis and macrophage apoptosis in murine atherosclerosis. Proc. Natl. Acad. Sci. U. S. A. 100 (18), 10423–10428. 10.1073/pnas.1732494100 12923293 PMC193577

[B22] Golabi P. Fukui N. Paik J. Sayiner M. Mishra A. Younossi Z. M. (2019). Mortality risk detected by atherosclerotic cardiovascular disease score in patients with nonalcoholic fatty liver disease. Hepatol. Commun. 3 (8), 1050–1060. 10.1002/hep4.1387 31388626 PMC6671783

[B23] Gupta A. Iadecola C. (2015). Impaired Aβ clearance: a potential link between atherosclerosis and Alzheimer’s disease. Front. Aging Neurosci. 7, 115. 10.3389/fnagi.2015.00115 26136682 PMC4468824

[B24] Hang L. Peng Y. Xiang R. Li X. Li Z. (2020). Ox-LDL causes endothelial cell injury through ASK1/NLRP3-mediated inflammasome activation via endoplasmic reticulum stress. Drug Des. Devel Ther. 14, 731–744. 10.2147/DDDT.S231916 PMC7047838 32158192

[B25] Herrington W. Lacey B. Sherliker P. Armitage J. Lewington S. (2016). Epidemiology of atherosclerosis and the potential to reduce the global burden of atherothrombotic disease. Circ. Res. 118 (4), 535–546. 10.1161/CIRCRESAHA.115.307611 26892956

[B26] Hetz C. (2012). The unfolded protein response: controlling cell fate decisions under ER stress and beyond. Nat. Rev. Mol. Cell Biol. 13 (2), 89–102. 10.1038/nrm3270 22251901

[B27] Hong J. Kim K. Kim J. H. Park Y. (2017). The role of endoplasmic reticulum stress in cardiovascular disease and exercise. Int. J. Vasc. Med. 2017, 2049217. 10.1155/2017/2049217 28875043 PMC5569752

[B28] Hotamisligil G. S. (2010a). Endoplasmic reticulum stress and the inflammatory basis of metabolic disease. Cell 140 (6), 900–917. 10.1016/j.cell.2010.02.034 20303879 PMC2887297

[B29] Hotamisligil G. S. (2010b). Endoplasmic reticulum stress and atherosclerosis. Nat. Med. 16 (4), 396–399. 10.1038/nm0410-396 20376052 PMC2897068

[B30] Ji C. Yi H. Huang J. Zhang W. Zheng M. (2021). Propofol alleviates inflammation and apoptosis in HCY-induced HUVECs by inhibiting endoplasmic reticulum stress. Mol. Med. Rep. 23 (5), 333. 10.3892/mmr.2021.11972 33760174 PMC7974316

[B31] Kattoor A. J. Goel A. Mehta J. L. (2019). LOX-1: regulation, signaling and its role in atherosclerosis. Antioxidants (Basel) 8 (7), 218. 10.3390/antiox8070218 31336709 PMC6680802

[B32] Ke D. Yan-zhu P. Xin Z. Ming-yan L. (2017). Progress of endoplasmic reticulum stress and macrophage M1/M2polarization. Adv. Anatomical Sci. Univ. 23 (02), 201–203+7. 10.16695/j.cnki.1006-2947.2017.02.025

[B33] Krüger-Genge A. Blocki A. Franke R. P. Jung F. (2019). Vascular endothelial cell Biology: an update. Int. J. Mol. Sci. 20 (18), 4411. 10.3390/ijms20184411 31500313 PMC6769656

[B34] Larroque-Cardoso P. Swiader A. Ingueneau C. Nègre-Salvayre A. Elbaz M. Reyland M. E. (2013). Role of protein kinase C δ in ER stress and apoptosis induced by oxidized LDL in human vascular smooth muscle cells. Cell Death Dis. 4 (2), e520. 10.1038/cddis.2013.47 23449456 PMC3734829

[B35] Lawrence de Koning A. B. Werstuck G. H. Zhou J. Austin R. C. (2003). Hyperhomocysteinemia and its role in the development of atherosclerosis. Clin. Biochem. 36 (6), 431–441. 10.1016/s0009-9120(03)00062-6 12951169

[B36] Lebeaupin C. Vallée D. Hazari Y. Hetz C. Chevet E. Bailly-Maitre B. (2018). Endoplasmic reticulum stress signalling and the pathogenesis of non-alcoholic fatty liver disease. J. Hepatol. 69 (4), 927–947. 10.1016/j.jhep.2018.06.008 29940269

[B37] Lee T. S. Lu T. M. Chen C. H. Guo B. C. Hsu C. P. (2021). Hyperuricemia induces endothelial dysfunction and accelerates atherosclerosis by disturbing the asymmetric dimethylarginine/dimethylarginine dimethylaminotransferase 2 pathway. Redox Biol. 46, 102108. 10.1016/j.redox.2021.102108 34438260 PMC8390558

[B38] Li Y. Schwabe R. F. DeVries-Seimon T. Yao P. M. Gerbod-Giannone M. C. Tall A. R. (2005). Free cholesterol-loaded macrophages are an abundant source of tumor necrosis factor-alpha and interleukin-6: model of NF-kappaB- and map kinase-dependent inflammation in advanced atherosclerosis. J. Biol. Chem. 280 (23), 21763–21772. 10.1074/jbc.M501759200 15826936

[B39] Libby P. Buring J. E. Badimon L. Hansson G. K. Deanfield J. Bittencourt M. S. (2019). Atherosclerosis. Nat. Rev. Dis. Prim. 5 (1), 56. 10.1038/s41572-019-0106-z 31420554

[B40] Lijun Y. Liangxiu H. Naxin L. (2019). A new approach to journal co-citation matrix construction based on the number of co-cited articles in journals. Scientometrics 120 (2), 507–517. 10.1007/s11192-019-03141-9

[B41] Lim W. S. Timmins J. M. Seimon T. A. Sadler A. Kolodgie F. D. Virmani R. (2008). Signal transducer and activator of transcription-1 is critical for apoptosis in macrophages subjected to endoplasmic reticulum stress *in vitro* and in advanced atherosclerotic lesions *in vivo* . Circulation 117 (7), 940–951. 10.1161/CIRCULATIONAHA.107.711275 18227389 PMC2276635

[B42] Lin H. Ni T. Zhang J. Meng L. Gao F. Pan S. (2018). Knockdown of Herp alleviates hyperhomocysteinemia mediated atherosclerosis through the inhibition of vascular smooth muscle cell phenotype switching. Int. J. Cardiol. 269, 242–249. 10.1016/j.ijcard.2018.07.043 30017525

[B43] Lindholm D. Korhonen L. Eriksson O. Kõks S. (2017). Recent insights into the role of unfolded protein response in ER stress in Health and disease. Front. Cell Dev. Biol. 5, 48. 10.3389/fcell.2017.00048 28540288 PMC5423914

[B44] Linna Z. Lifeng Z. (2017). Pioglitazone attenuates the effect of insulin resistance on carotid atherosclerosis in patients with type 2 diabetes mellitus. J. Clin. Ration. Drug Use 10 (36), 47–48. 10.15887/j.cnki.13-1389/r.2017.36.023

[B45] Liu M. Q. Chen Z. Chen L. X. (2016). Endoplasmic reticulum stress: a novel mechanism and therapeutic target for cardiovascular diseases. Acta Pharmacol. Sin. 37 (4), 425–443. 10.1038/aps.2015.145 26838072 PMC4820795

[B46] Liu X. Hou D. Lin F. Luo J. Xie J. Wang Y. (2019). The role of neurovascular unit damage in the occurrence and development of Alzheimer’s disease. Rev. Neurosci. 30 (5), 477–484. 10.1515/revneuro-2018-0056 30530893

[B47] Lubrano V. Balzan S. (2014). LOX-1 and ROS, inseparable factors in the process of endothelial damage. Free Radic. Res. 48 (8), 841–848. 10.3109/10715762.2014.929122 24886290

[B48] Matthews K. A. Xu W. Gaglioti A. H. Holt J. B. Croft J. B. Mack D. (2019). Racial and ethnic estimates of Alzheimer’s disease and related dementias in the United States (2015-2060) in adults aged ≥65 years. Alzheimers Dement. 15 (1), 17–24. 10.1016/j.jalz.2018.06.3063 30243772 PMC6333531

[B49] Milutinović A. Šuput D. Zorc-Pleskovič R. (2020). Pathogenesis of atherosclerosis in the tunica intima, media, and adventitia of coronary arteries: an updated review. Bosn. J. Basic Med. Sci. 20 (1), 21–30. 10.17305/bjbms.2019.4320 31465719 PMC7029210

[B50] Moore K. J. Tabas I. (2011). Macrophages in the pathogenesis of atherosclerosis. Cell 145 (3), 341–355. 10.1016/j.cell.2011.04.005 21529710 PMC3111065

[B51] Myoishi M. Hao H. Minamino T. Watanabe K. Nishihira K. Hatakeyama K. (2007). Increased endoplasmic reticulum stress in atherosclerotic plaques associated with acute coronary syndrome. Circulation 116 (11), 1226–1233. 10.1161/CIRCULATIONAHA.106.682054 17709641

[B52] Nebel R. A. Aggarwal N. T. Barnes L. L. Gallagher A. Goldstein J. M. Kantarci K. (2018). Understanding the impact of sex and gender in Alzheimer’s disease: a call to action. Alzheimers Dement. 14 (9), 1171–1183. 10.1016/j.jalz.2018.04.008 29907423 PMC6400070

[B53] Oakes S. A. Papa F. R. (2015). The role of endoplasmic reticulum stress in human pathology. Annu. Rev. Pathol. 10, 173–194. 10.1146/annurev-pathol-012513-104649 25387057 PMC5568783

[B54] Oh J. Riek A. E. Weng S. Petty M. Kim D. Colonna M. (2012). Endoplasmic reticulum stress controls M2 macrophage differentiation and foam cell formation. J. Biol. Chem. 287 (15), 11629–11641. 10.1074/jbc.M111.338673 22356914 PMC3320912

[B74] Organization W. H. (2011). Who web site on cardiovascular diseases: strategic priorities, fact sheets, world health day 2013, publications.

[B55] Pisto P. Santaniemi M. Bloigu R. Ukkola O. Kesäniemi Y. A. (2014). Fatty liver predicts the risk for cardiovascular events in middle-aged population: a population-based cohort study. BMJ Open 4 (3), e004973. 10.1136/bmjopen-2014-004973 PMC3963104 24650811

[B56] Ren J. Bi Y. Sowers J. R. Hetz C. Zhang Y. (2021). Endoplasmic reticulum stress and unfolded protein response in cardiovascular diseases. Nat. Rev. Cardiol. 18 (7), 499–521. 10.1038/s41569-021-00511-w 33619348

[B57] Ron D. Walter P. (2007). Signal integration in the endoplasmic reticulum unfolded protein response. Nat. Rev. Mol. Cell Biol. 8 (7), 519–529. 10.1038/nrm2199 17565364

[B58] Rondanelli M. Perna S. Peroni G. Guido D. (2016). A bibliometric study of scientific literature in Scopus on botanicals for treatment of androgenetic alopecia. J. Cosmet. Dermatol 15 (2), 120–130. 10.1111/jocd.12198 26608588

[B59] Salminen A. Kaarniranta K. Kauppinen A. (2020). ER stress activates immunosuppressive network: implications for aging and Alzheimer’s disease. J. Mol. Med. Berl. 98 (5), 633–650. 10.1007/s00109-020-01904-z 32279085 PMC7220864

[B60] Schmitz M. L. Shaban M. S. Albert B. V. Gökçen A. Kracht M. (2018). The crosstalk of endoplasmic reticulum (ER) stress pathways with NF-κB: complex mechanisms relevant for cancer, inflammation and infection. Biomedicines 6 (2), 58. 10.3390/biomedicines6020058 29772680 PMC6027367

[B61] Shio H. Haley N. J. Fowler S. (1979). Characterization of lipid-laden aortic cells from cholesterol-fed rabbits. III. Intracellular localization of cholesterol and cholesteryl ester. Lab. Invest. 41 (2), 160–167. 459432

[B62] Small D. M. Bond M. G. Waugh D. Prack M. Sawyer J. K. (1984). Physicochemical and histological changes in the arterial wall of nonhuman primates during progression and regression of atherosclerosis. J. Clin. Invest. 73 (6), 1590–1605. 10.1172/JCI111366 6725553 PMC437070

[B63] Suzuki H. Kurihara Y. Takeya M. Kamada N. Kataoka M. Jishage K. (1997). A role for macrophage scavenger receptors in atherosclerosis and susceptibility to infection. Nature 386 (6622), 292–296. 10.1038/386292a0 9069289

[B64] Tabas I. (2000). Cholesterol and phospholipid metabolism in macrophages. Biochim. Biophys. Acta 1529 (1-3), 164–174. 10.1016/s1388-1981(00)00146-3 11111086

[B65] Tabas I. (2005). Consequences and therapeutic implications of macrophage apoptosis in atherosclerosis: the importance of lesion stage and phagocytic efficiency. Arterioscler. Thromb. Vasc. Biol. 25 (11), 2255–2264. 10.1161/01.ATV.0000184783.04864.9f 16141399

[B66] Tabas I. (2010). The role of endoplasmic reticulum stress in the progression of atherosclerosis. Circ. Res. 107 (7), 839–850. 10.1161/CIRCRESAHA.110.224766 20884885 PMC2951143

[B67] Tabas I. Ron D. (2011). Integrating the mechanisms of apoptosis induced by endoplasmic reticulum stress. Nat. Cell Biol. 13 (3), 184–190. 10.1038/ncb0311-184 21364565 PMC3107571

[B68] Takousis P. Sadlon A. Schulz J. Wohlers I. Dobricic V. Middleton L. (2019). Differential expression of microRNAs in Alzheimer’s disease brain, blood, and cerebrospinal fluid. Alzheimers Dement. 15 (11), 1468–1477. 10.1016/j.jalz.2019.06.4952 31495604

[B69] Targher G. Byrne C. D. Lonardo A. Zoppini G. Barbui C. (2016). Non-alcoholic fatty liver disease and risk of incident cardiovascular disease: a meta-analysis. J. Hepatol. 65 (3), 589–600. 10.1016/j.jhep.2016.05.013 27212244

[B70] Thorp E. Li G. Seimon T. A. Kuriakose G. Ron D. Tabas I. (2009). Reduced apoptosis and plaque necrosis in advanced atherosclerotic lesions of Apoe-/- and Ldlr-/- mice lacking CHOP. Cell Metab. 9 (5), 474–481. 10.1016/j.cmet.2009.03.003 19416717 PMC2695925

[B71] Tian S. Li B. Lei P. Yang X. Zhang X. Bao Y. (2018). Sulforaphane improves abnormal lipid metabolism via both ERS-dependent XBP1/ACC &SCD1 and ERS-independent SREBP/FAS pathways. Mol. Nutr. Food Res. 62 (6), e1700737. 10.1002/mnfr.201700737 29380937

[B72] Toyomasu K. Adachi H. Enomoto M. Fukami A. Nakamura S. Nohara Y. (2021). Impact of combined elevations of homocysteine and asymmetric dimethylarginine on all-cause death - the Tanushimaru Study. J. Cardiol. 78 (2), 129–135. 10.1016/j.jjcc.2021.01.011 33551145

[B73] van Eck N. J. Waltman L. (2010). Software survey: VOSviewer, a computer program for bibliometric mapping. Scientometrics 84 (2), 523–538. 10.1007/s11192-009-0146-3 20585380 PMC2883932

[B75] Wu H. Chen Z. Chen J. Z. Pei L. G. Xie J. Wei Z. H. (2018). High mobility group B-1 (HMGB-1) promotes apoptosis of macrophage-derived foam cells by inducing endoplasmic reticulum stress. Cell Physiol. Biochem. 48 (3), 1019–1029. 10.1159/000491970 30041247

[B76] Yan L. (2005). Inflammation, insulin resistance common to type 2 diabetes and atherosclerosis. Foreign Med. Endocrinol. (03), 150–152.

[B77] Yang S. Wu M. Li X. Zhao R. Zhao Y. Liu L. (2020). Role of endoplasmic reticulum stress in atherosclerosis and its potential as a therapeutic target. Oxid. Med. Cell Longev. 2020, 9270107. 10.1155/2020/9270107 32963706 PMC7499294

[B78] Yao S. Miao C. Tian H. Sang H. Yang N. Jiao P. (2014a). Endoplasmic reticulum stress promotes macrophage-derived foam cell formation by up-regulating cluster of differentiation 36 (CD36) expression. J. Biol. Chem. 289 (7), 4032–4042. 10.1074/jbc.M113.524512 24366867 PMC3924270

[B79] Yao S. Yang N. Song G. Sang H. Tian H. Miao C. (2012). Minimally modified low-density lipoprotein induces macrophage endoplasmic reticulum stress via toll-like receptor 4. Biochim. Biophys. Acta 1821 (7), 954–963. 10.1016/j.bbalip.2012.03.003 22480542

[B80] Yao S. T. Zhao L. Miao C. Tian H. Yang N. N. Guo S. D. (2014b). Endoplasmic reticulum stress mediates oxidized low density lipoprotein-induced scavenger receptor A1 upregulation in macrophages. Sheng Li Xue Bao 66 (5), 612–618. 10.1074/jbc.M113.524512 25332008

[B81] Yue C. Chao-mei C. Ze-yuan L. Zhi-gang H. Xian-wen W. (2015). The methodology function of Cite Space mapping knowledge domains. Sci. Res. 33 (02), 242–253. 10.16192/j.cnki.1003-2053.2015.02.009

[B82] Zhou J. Lhoták S. Hilditch B. A. Austin R. C. (2005). Activation of the unfolded protein response occurs at all stages of atherosclerotic lesion development in apolipoprotein E-deficient mice. Circulation 111 (14), 1814–1821. 10.1161/01.CIR.0000160864.31351.C1 15809369

[B83] Zhu G. Lee A. S. (2015). Role of the unfolded protein response, GRP78 and GRP94 in organ homeostasis. J. Cell Physiol. 230 (7), 1413–1420. 10.1002/jcp.24923 25546813 PMC4725317

